# Scaling laws for lattice distortions: Application to high entropy alloys

**DOI:** 10.1093/pnasnexus/pgae117

**Published:** 2024-03-18

**Authors:** Zhaowei Wang, A S L Subrahmanyam Pattamatta, Jian Han, David J Srolovitz

**Affiliations:** Department of Materials Science and Engineering, City University of Hong Kong, Hong Kong SAR 999077, China; Department of Mechanical Engineering, The University of Hong Kong, Pokfulam Road, Hong Kong SAR 999077, China; Department of Materials Science and Engineering, City University of Hong Kong, Hong Kong SAR 999077, China; Department of Mechanical Engineering, The University of Hong Kong, Pokfulam Road, Hong Kong SAR 999077, China; Greater Bay Joint Division, Shenyang National Laboratory for Materials Science, Hong Kong SAR 999077, China

## Abstract

Lattice distortions are intrinsic features of all solid solution alloys associated with varying atomic radii; this phenomenon facilitates the formation of single-phase solid solutions. Using high-entropy alloys (HEAs), as an example, we investigate the influence of variations in inter-atomic separations for stabilizing and controlling their structural, mechanical, and thermodynamic properties. This is done through a combination of statistical mechanics analysis and molecular dynamics simulations on simplified 2D systems, as well as a 3D crystals with harmonic and anharmonic inter-atomic bonds with varying natural inter-atomic separations. We demonstrate that the impact of this inter-atomic length disorder (representing static lattice distortion) and temperature fluctuations (representing dynamic lattice distortion) on fundamental and universal thermodynamic, structural, and elastic characteristics are similar and can be unified through effective temperature; i.e. a scaling law for HEAs that establishes a relationship between these factors. This scaling law reveals that different HEAs (i.e. varying degrees of local lattice distortions) collapse onto a single curve when plotted against the effective temperature. We demonstrate that lattice distortion significantly enhances the stability of solid solution alloys (relative to phase separation or ordering by effectively increasing the temperature of the system; this stabilization effect is particularly pronounced in HEAs).

**Significance Statement**

Compositionally induced atomic spacing fluctuations enhance the thermodynamic stability of multiprincipal component alloys. This is demonstrated through rigorous statistical mechanics of low-dimensional models and validated in 3D materials via classical molecular dynamics. We identify a robust scaling law that formally connects static bond length and thermal vibration-induced (dynamic) dispersion.

## Introduction

High entropy alloys (HEAs) have garnered significant attention in recent years due to their fascinating range of physical and mechanical properties (e.g. see Refs (1–4)). These exceptional properties have been achieved by expanding the composition palette far beyond that of most conventional alloys. HEAs are often described as materials with nearly equimolar compositions of five or more principal elemental components (5). The term “high entropy” is associated with the observation that the entropy of mixing in an ideal, random solid solution increases with the number of components (*n*) according to the equation $\Delta S_{m}=-k_{B}\sum_{i}c_{i}\ln\;c_{i}$, where $c_{i}$ represents the atomic fraction of component *i*, the sum is over all *n* components, $k_{B}$ is the Boltzmann constant. This expression reduces to $\Delta S_{m}=k_{B}\ln\;n$ for an equimolar alloy. The increase in the entropy of mixing with *n* contributes to the enhanced stability of the solid solution phase, reducing the tendency for formation of multiphase or ordered structures.

The entropy of an HEA encompasses contributions that extend beyond the straightforward entropy of mixing, including vibrational, electronic, and magnetic components (6). We note that due to the differing atomic sizes of individual alloy components, the atoms in an HEA do not sit on ideal lattice sites (defined by the Bravais lattice and basis vectors). Hence, the variation in atomic size and deviation of atom locations from the ideal lattice in a random solid solution provides an additional contribution to the entropy, which we denote noncomposition entropy $\Delta S_{\mathrm{nc}}$. (This noncomposition entropy is distinct from the entropy of mixing $\Delta S_{m}$.) In a single-component glassy system, $\Delta S_{\mathrm{nc}}$ is referred to as “configurational” or “information” entropy or “complexity” (7). Random local lattice distortions (misfit) are responsible, in part, for the high strength of high entropy alloys (e.g. see Refs (8–11)). Here, we focus on the thermodynamic implications of such random distortions.

The effects of atomic-size-induced distortions on structure and properties are difficult to assess in real alloy systems since these are necessarily convoluted with variations in bonding between different types of atoms (12). One approach for simplifying the relationships between atom types and their bonding effects is to use average potentials, such as the average-atom embedded-atomic method (EAM) potential (13). However, this mean-field approach has limitations; it does not account for the influences of lattice distortion or atomic displacement and thus $\Delta S_{\mathrm{nc}}$ (14).

In this article, our objective is to disentangle these effects and specifically isolate the influence of atomic size-induced distortions in HEAs. To accomplish this, we concentrate on a theoretical model system, where all bond strengths are uniform, while variations in bond lengths persist. The general expression for the potential energy *Φ* of such a bond stretched to a length *L* from its natural length $L_{0}$ is given by, $\Phi =\frac{1}{2!}K_{2}(L-L_{0}{)}^{2}+\frac{1}{3!}K_{3}(L-L_{0}{)}^{3}+$  $\frac{1}{4!}K_{4}(L-L_{0}{)}^{4}+\cdots$. We normalize this expression by dividing both sides with $K_{2}L_{0}^{2}$ (dimensions of energy), yielding:


(1) $$\phi =\frac{1}{2!}{\left(\ell -1\right)}^{2}+\frac{1}{3!}k_{3}{\left(\ell -1\right)}^{3}+\frac{1}{4!}k_{4}{\left(\ell -1\right)}^{4}+\cdots ,$$

where $\phi =\Phi /(K_{2}L_{0}^{2})$, $\ell =L/L_{0}$, and $k_{n}=K_{n}L_{0}^{n-2}/K_{2}$ are the normalized quantities (see Table S1 for relevant normalized quantities).

Furthermore, to minimize the impact of bonding intricacies, we simplify our approach by idealizing atomic bonding as purely harmonic, i.e. set $k_{n}$ to zero for n>2. In fact, spring (harmonic-bonded) models have been utilized in various studies to investigate structural and mechanical properties for many years (15–20). Barron and Stacey (15, 17, 18) demonstrated that harmonic bond lengths are unaltered by heating, whereas harmonic-bonded crystals contract. Toda-Caraballo (19) employed both density functional theory (DFT) and a spring model to predict lattice displacements in HEAs, using synthetic pair potentials. Liu (20) introduced a method to manipulate disorder in an unstressed spring network through bond-stiffness disorder; randomly distorting an initially close-packed lattice structure showed that the network becomes auxetic. Of course, real materials are never truly harmonic and the anharmonic effects can be introduced by preserving the higher order stiffness terms $k_{n},n>2$ in Eq. 1.

We note that thermal vibrations and random atomic size effects share an important feature (6, 21–23). Much like “snapshots” of a crystal at finite temperatures show that atom positions fluctuate from their ideal lattice positions, a crystal that is randomly populated by atoms of varying sizes also exhibits atoms displaced from ideal lattice sites at 0 K. This structural analogy has been widely discussed in the literature (24–27). These discussions commonly focus on how both dynamic and static components contribute to the final atomic displacements from their ideal lattice positions. The observation that vibrational contributions to the free energy can be described by examining a time average of “snapshots” implies a fundamental connection between vibrational and noncompositional entropy. This connection, in turn, suggests an intimate relationship between lattice distortions in HEAs and their thermal behavior. Here, we examine the effect of this correlation between static lattice distortions and dynamic thermal vibrations on thermodynamic behavior.

In our atomic bonding model, we specifically narrow our focus to examine the influence of atomic bond length disorder, rather than the dispersion of atomic sizes. Although an *n*-component HEA possesses n(n+1)/2 distinct equilibrium bond lengths, in our model, we treat atomic size dispersion as a continuous and smooth bond length distribution, as firstly outlined in Refs (28, 29). We hypothesize that this relationship could potentially be formally articulated using the concept of dual lattices, which is often employed in lattice spin models. However, it is important to note that we have not pursued such a derivation. Consequently, in the subsequent sections of this article, we frequently refer to bond length disorder, which is a broader term than atomic size variation and provides a more comprehensive representation of alloy systems. Leveraging the analogy between vibrational effects and bond length disorder, we aim to predict fundamental properties of HEAs, including thermal expansion and the temperature-dependent behavior of elastic constants.

## Results

### Thermal behavior of a monatomic, harmonic-bond material

Before delving into the complexities of HEAs, we revisit the thermal behavior of simpler, single-component systems. As previously mentioned, understanding the thermal behavior of a single-component system sheds light on atomic size disorder in HEAs. We start by analyzing our investigation to a 2D system. These low-dimensional models are chosen for their amenability to rigorous theoretical analysis. Eventually, we apply our insights to a 3D crystal structure. In essence, we substitute a lattice of many atoms with varying bond lengths for an ensemble of smaller 2D diatomic models. This allows us to focus on ensemble averages rather than volumetric averages. While our approach is grounded in established principles of statistical mechanics, our primary aim here is to empirically demonstrate its validity. Detailed derivations and key findings for these simplified systems are provided in the Supplementary material.

#### 2D: a harmonic-bond *Λ* model

**Fig. 1.**
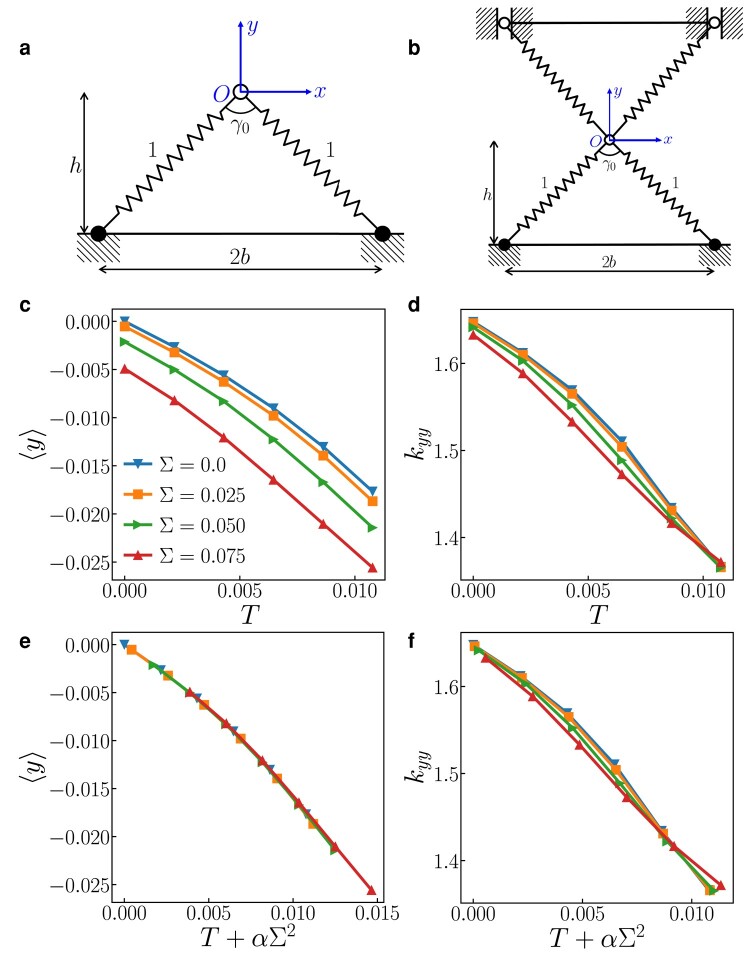
Schematic of a) a symmetric *Λ* model and b) a centro-symmetric *Λ* model. The vertex atom is located at the origin. All geometric parameters are labeled in dimensionless forms. c) The vertex height ⟨y⟩ and d) vertical stiffness $k_{yy}$ vs. temperature *T* for different bond-length dispersions *Σ*. e) Vertex height ⟨y⟩ and f) vertical stiffness $k_{yy}$ vs. effective temperature $T_{\mathrm{eff}}=T+\alpha \Sigma^{2}$. The results for the *Λ* model and its centro-symmetric version are equivalent.

The “*Λ*” model is designed to capture bond angle effects to represent an essential feature of crystal lattices; see Fig. 1(a). The *Λ* configuration is composed of a single “free” atom, bonded to two fixed atoms via harmonic springs. Here, we fix both the nondimensional spring stiffnesses and nondimensional unstretched spring length to 1.

The potential energy for the free atom at r=(x,y) is


(2) $$\begin{array}{rl} U(r) & =\frac{1}{2}\{{\left[\sqrt{(x+b{)}^{2}+(y+h{)}^{2}}-1\right]}^{2}\\ & \;+{\left[\sqrt{(x-b{)}^{2}+(y+h{)}^{2}}-1\right]}^{2}\}. \end{array}$$

where *h*, *b*, and $\gamma_{0}$ are as in Fig. 1(a). The associated partition function is $Z=(2\pi /\beta )\int e^{-\beta U}dr\approx \pi /(\beta bh)$ (see derivation of Eq. S9).

Expanding the potential energy about r=0 to the third order and the displacement in the partition function about r=0 to the fifth order (see derivation of S(S12)), we find the averaged position of the free (vertex) atom:


(3) $$\langle x\rangle^{0}=0,\;\langle y\rangle^{0}=-\frac{2b^{4}+(b^{2}-h^{2}{)}^{2}}{4b^{2}h^{3}}T.$$

This result shows that the average position of the free atom shifts towards the base (analogous to a reduction of lattice parameter) with increasing temperature and the magnitude of the shift is directly proportional to temperature (at low temperature). The details of the calculations and the energy landscape are presented in Fig. S1. This finite-temperature contraction ($\langle y\rangle^{0}\leq 0$) is consistent with the energy landscape.

It is important to note that the thermal contraction observed in the *Λ* model is not primarily associated with its lack of centro-symmetry. Consider the centro-symmetric double-*Λ* model Fig. 1(b) (where the base atoms are free to move in the vertical direction). Examination of this figure shows that horizontal (thermal) vibrations of the free vertex atom will induce a downward motion of the two upper base nodes/atoms; i.e. thermal contraction. Hence, thermal contraction is not associated with the asymmetry of the basic *Λ* model—rather it is a generic feature of such a 2D model (in fact for all dimensions, d≥2). This is clearly shown below for the centro-symmetric harmonic FCC lattice.

**Fig. 2.**
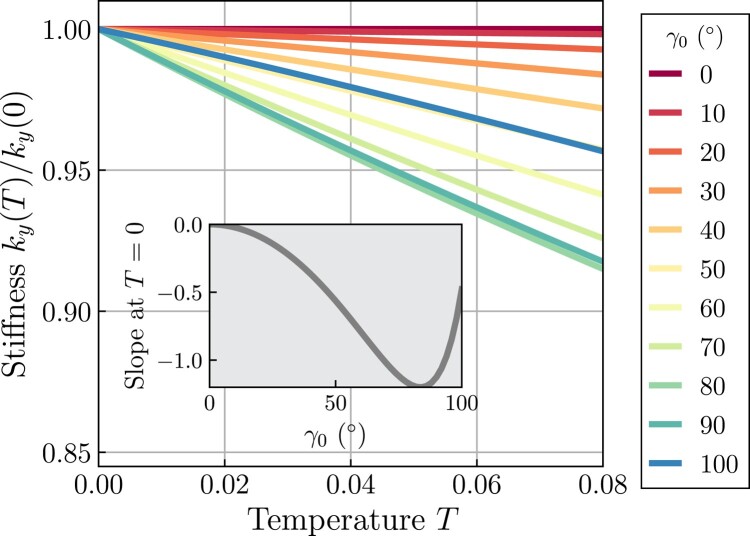
Temperature dependence of the stiffness of the *Λ* model for several different bond angles $\gamma_{0}$ based upon Eq. 4. The inset shows the $\gamma_{0}$-dependence of the slope at T=0, $[d(k_{y}(T)/k_{y}(0))/d\gamma_{0}{]}_{T=0}$.

We now focus on the temperature dependence of the stiffness in the *Λ* model in the *y*-direction (i.e. x=0). The inverse stiffness for the *Λ* model is $k_{y}^{-1}=(\partial \langle y\rangle^{0}/\partial f{)}_{\;f=0}$, where *f* is a force on the atom in the *y*-direction. To obtain an explicit form of the temperature dependence of the stiffness (in the *y*-direction), we expand the potential for small displacements (see derivative of Eq. S27) and obtain


(4) $$k_{y}(T)=\frac{h^{2}{\left[16-3{\hat{b}}^{2}({\hat{b}}^{2}-4)T\right]}^{2}}{128-288{\hat{b}}^{2}({\hat{b}}^{2}-2)T+(45/2){\hat{b}}^{4}({\hat{b}}^{2}-4{)}^{2}T^{2}},$$

where $\hat{b}\equiv b/h=\tan\;(\gamma_{0}/2)$ and $\gamma_{0}$ is the bond angle (Fig. 1(a)). The temperature dependence of the stiffness is shown in Fig. 2 for several bond angles. For all $\gamma_{0}>0$, the stiffness decreases with increasing temperature. This is associated with the displacement of the free atom mean position toward negative *y* with increasing temperature and the curvature of the potential energy landscape decreases with decreasing *y* (see Fig. S1). The stiffness reaches the minimum at $\gamma_{0}=2\tan^{-1}\;(2/\sqrt{5})=83.62^{\circ }$.

To validate the approximate theoretical predictions for the *Λ* model, we conduct a comparison between the predictions of the temperature dependence of the lattice parameter (Eq. 3) and the stiffness (Eq. 4) with the results obtained from MD simulations, as illustrated in Fig. S3. Remarkably, we observe excellent agreement at lower temperatures, although deviations became more pronounced at higher temperatures due to the inherent limitations of the small-displacement expansion employed in our analysis.

These results signify that, even in the scenario where bonding is perfectly harmonic but the bond angle $\gamma_{0}$ deviates from $180^{\circ }$, the *Λ* model experiences contraction and a reduction in the elastic constant as the temperature increases. Notably, a significant portion of the elastic constant’s softening as temperature rises can be attributed to thermal contraction. An examination of 4 reveals that, to the leading order, the elastic constant scales with the elevation of the free atom above its base as $h^{2}$, with dh/dT<0.

#### 3D: a harmonic-bond FCC crystal

We next turn our attention to the temperature-dependent properties of a 3D FCC crystal with harmonic bonds. Due to the increased geometric complexity inherent in a periodic 3D lattice—as opposed to the simpler diatomic and *Λ* models—we employ MD simulations (see Methods) to ascertain how temperature influences the lattice parameter $a^{0}$ and the elastic constants $C_{ij}^{0}$.

**Fig. 3.**
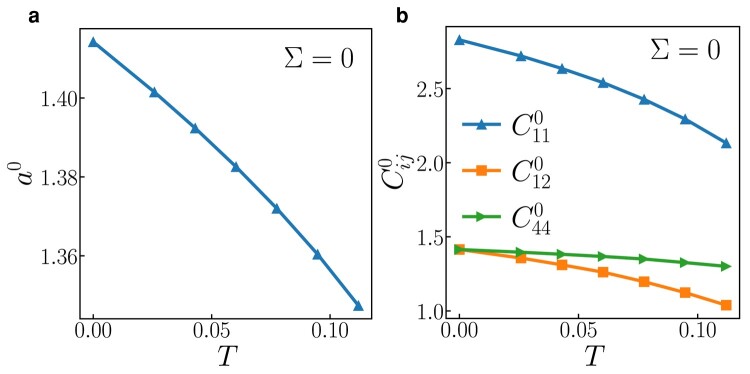
a) Lattice parameter $a^{0}$ vs. temperature. b) Elastic constants $C_{11}^{0}$, $C_{12}^{0}$, and $C_{44}^{0}$ vs. temperature.

In Fig. 3(a), it is evident that the time-averaged cubic lattice parameter $a^{0}$ exhibits a decreasing trend as the temperature rises, as documented in Ref. (30). Figure 3(b) showcases a corresponding behavior, where all three cubic elastic constants ($C_{11}^{0}$, $C_{12}^{0}$, $C_{44}^{0}$) also decrease with increasing temperature. These observations of contraction and softening in the elastic constants as temperature increases align seamlessly with the preceding analysis of the temperature dependence within the 2D *Λ* model. Note that existence of thermal contraction of the harmonic 3D FCC model is insensitive to model details (including second neighbors or not), as shown in the Fig. S7. It should be noted that the thermal expansion coefficient is nonzero near 0 K (in violation of the third law of thermodynamics), like in all classical (not quantum) MD simulations (31–33).

In the 2D *Λ* model, the bond angle undergoes temperature-dependent variations, which are associated with thermal contraction. In contrast, within the 3D FCC lattice, there is no alteration in the mean bond angle due to the presence of inversion symmetry within the FCC crystal structure. Nevertheless, the FCC lattice still experiences contraction as it is heated. This may be deduced from the simple, classical arguments of Stacey and Barron (15, 17) who conclude that transverse thermal vibrations lead to negative thermal expansion. This contraction scales proportionally to the square of the amplitude of the thermal vibrations, like say a $\Sigma^{2}$. The amplitude, in turn, is directly proportional to the square root of the temperature, implying that, to the leading order, the contraction is linearly related to temperature—a behavior evident in Fig. 3(a).

Contrary to most materials, whose elastic constants decrease due to the anharmonicity of the crystal as temperature increases, our harmonic FCC crystal exhibits an interesting behavior: it contracts upon heating, but its elastic constants also decrease (34, 35). This seemingly paradoxical behavior can be understood by examining the variation in potential energy with applied strain, as detailed in Fig. S4. Elastic constants are directly linked to the second derivative of the potential energy with respect to strain. To leading order, this second derivative shows a linear decrease with compression. Given that the 3D lattice contracts upon heating, it is logical that the elastic constants would also decrease. This trend is consistent with our MD results (Fig. 3). The asymmetric nature of the potential energy curve (Fig. S4) suggests that thermal vibrations cause the thermal contraction; bonds are more easily compressed than stretched and spend more time in a compressed state. This asymmetric behavior, particularly the increased curvature with rising $\varepsilon_{xx}$, accounts for the decreasing trend in $C_{11}$ as the system heats up and contracts (i.e. negative $\varepsilon_{xx}$).

### A “multi”component harmonic-bond material

We introduce a simple, generic model for a high entropy alloy in which bonds between nearest neighbor atoms share the same stiffness, are harmonic, and have unstretched bond lengths chosen at random from a Gaussian distribution:


(5) $$P(\ell_{0})=\frac{1}{\Sigma \sqrt{2\pi }}\text{exp}\left[-\frac{(\ell_{0}-1{)}^{2}}{2\Sigma^{2}}\right],$$

where the bonds have equilibrium lengths $L_{0}$ distributed about a mean unstretched bond length $L^{0}$, $\ell_{0}\equiv L_{0}/L^{0}$ and the standard deviation of bond length distribution is *Σ*. This is a continuous version of an HEA with ideal bonds. While greatly simplified, this model allows us to focus solely on the effect of randomness in atomic size in a model amenable to analysis.

In the majority of solid solution HEAs, the atom sizes tend to exhibit close clustering. For instance, in extensively studied alloys like Cantor CrMnFeCoNi (6, 36, 37), the standard deviation in atomic sizes or bond lengths typically remains below 5%. It is noteworthy that at a finite temperature, even a single-component (harmonic) system also features a Gaussian distribution of instantaneous bond lengths. However, in this case, the standard deviation scales with temperature (following a $T^{1/2}$ relationship) rather than being influenced by local composition variations.

#### 2D: ensemble of random harmonic-bond *Λ* models

We first study a 2D model that captures bond angle effects present in crystal lattices; see Fig. 1(a). The *Λ* configuration is composed of a single free vertex atom, bonded to two fixed atoms via harmonic springs. Here, we fix the nondimensional stiffnesses of both bonds and choose each of the two unstretched bond lengths ($\ell_{1}$ and $\ell_{2}$) at random from the Gaussian distribution, Eq. 5. The vertex atom located at r corresponds to the particular unstretched bond lengths $\ell_{1}$ and $\ell_{2}$. The probability that the vertex atom is at r is


(6) $$P(r)=P(\ell_{1}(r))P(\ell_{2}(r))\equiv \frac{e^{-\beta U_{\mathrm{eff}}(r)}}{Z_{\mathrm{eff}}},$$

where *β* is the inverse temperature, and we introduced an effective potential $U_{\mathrm{eff}}(r)$ and partition function $Z_{\mathrm{eff}}$. This effective potential energy is


(7) $$\begin{array}{rl} U_{\mathrm{eff}}(r) & =\frac{1}{2\beta \Sigma^{2}}\{{\left[\sqrt{(x+b{)}^{2}+(y+h{)}^{2}}-1\right]}^{2}\\ & \;+{\left[\sqrt{(x-b{)}^{2}+(y+h{)}^{2}}-1\right]}^{2}\}+\frac{1}{\beta }\ln\;\left(\frac{2\pi \Sigma^{2}}{Z_{\mathrm{eff}}}\right), \end{array}$$

where *b* and *h* describe the geometry of the *Λ* model (see Fig. 1(a)). By comparison with the case without bond length disorder ($U(r)=\frac{1}{2}\{[\sqrt{(x+b{)}^{2}+(y+h{)}^{2}}-1{]}^{2}+[\sqrt{(x-b{)}^{2}+(y+h{)}^{2}}-1{]}^{2}\}$), we find that the finite-temperature, uniform bond length *Λ* model has the same energy as the ensemble averaged (random bond length) model if we set $\Sigma^{2}=\beta^{-1}=T$. Since $U_{\mathrm{eff}}(r)$ differs from U(r) by a term independent of r, the equilibrium positions/standard deviations from $U_{\mathrm{eff}}(r)$ and U(r) are the same. So, the disordered system can be described as identical to the uniform-bond-length *Λ* model at effective temperature: $T_{\mathrm{eff}}=T+\Sigma^{2}$ (see derivation of Eq. S34).

We conduct MD simulations to ascertain the ensemble-averaged thermal contraction and stiffness properties of the *Λ* model, examining their dependencies on both the standard deviation in bond lengths and temperature. These results are illustrated in Fig. 1. Similar to the behavior observed in the uniform-bond-length model, we observe that the vertex height ⟨y⟩ and the stiffness in the *y*-direction, denoted as $k_{yy}$, exhibit a decrease as temperature increases. Analyzing the temperature dependencies of both the vertex atom position ⟨y⟩ and $k_{yy}$, it is evident that altering the standard deviation in unstretched bond lengths primarily shifts the curves to higher temperatures. However, this effect is notably weaker for $k_{yy}$ and is not observed at the highest temperatures, as illustrated in Fig. 1(d).

Comparison of the MD data for ⟨y⟩ and $k_{yy}$ with the proposed


(8) $$T_{\mathrm{eff}}=T+\alpha \Sigma^{2},$$

(where *α* is a constant) show two main features. Firstly, this scaling approach effectively collapses all lattice parameters and stiffness properties as functions of both *T* and *Σ* onto single curves, as vividly demonstrated in Fig. 1(d) and (e). The collapse is exceptionally close to perfect for lattice parameters and outstanding for stiffness, particularly at low temperatures. Secondly, it is noteworthy that the constant *α* is not equal to 1, as evident in Fig. S5. Moreover, *α* takes on distinct values for lattice parameters (α=0.69) and stiffness (α=0.10), emphasizing the differentiation between these properties.

As derived earlier, it is apparent that α=1 results in a poor agreement for both the vertex height and stiffness, as depicted in Fig. S5. The data strongly indicate that both ⟨y⟩ and $k_{yy}$ exhibit scaling behavior with respect to both *T* and $\Sigma^{2}$. However, it is important to note that the scaling factors for these two properties are not identical. In essence, the effective temperature scales with both *T* and $\Sigma^{2}$, with *α* serving as the scaling factor. The figures in Fig. 1(d) and (e) clearly demonstrate the accuracy of this scaling relationship for ⟨y⟩ and $k_{yy}$ concerning *T* and $\Sigma^{2}$. Notably, *α* values that yield the best fit to ⟨y⟩ and $k_{yy}$ are found to be 0.69 and 0.10, respectively.

Concurrently, it is important to note that the derivation of thermal contraction is not the primary focus of our study. Instead, our emphasis lies in utilizing a simplified *Λ* model to derive the fundamental scaling law for lattice distortion, which constitutes our most significant finding.

#### 3D: random harmonic-bond FCC crystal

**Fig. 4.**
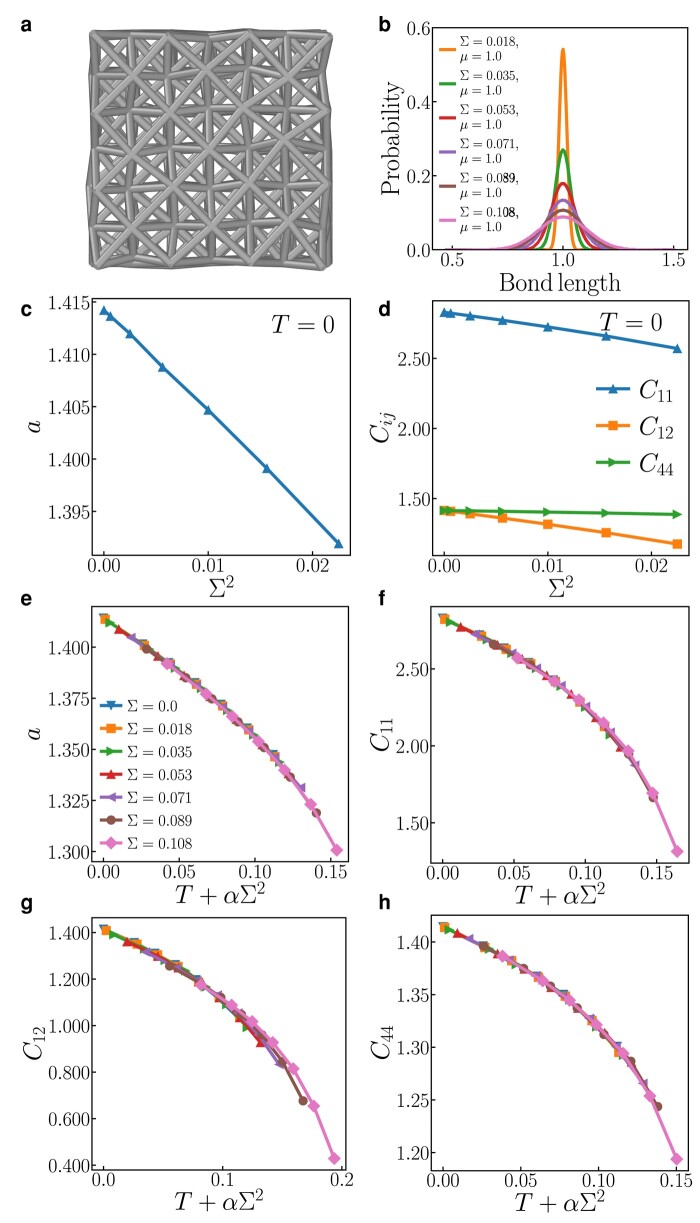
a) Distorted harmonically bonded FCC crystal (a part of big sample obtained by MD) for Σ=0.071. b) Bond-length distribution after relaxation; the legend shows the values of its standard deviation *Σ*. c) The mean lattice parameter and b) elastic constants vs. $\Sigma^{2}$ at T=0 and (c)–(f) vs. $T_{\mathrm{eff}}=T+\alpha \Sigma^{2}$ for several values of *Σ*. The best fit values of *α* are 3.6, 4.5, 7.0, and 3.3 for *a*, $C_{11}$, $C_{12}$, and $C_{44}$, respectively.

We conduct atomistic simulations, encompassing both static relaxation and MD, to delve into a harmonically bonded FCC crystal where unstretched bond lengths are randomly drawn in accordance with Eq. 5. The resultant relaxed structure at 0 K is presented in Fig. 4(a), while Fig. 4(b) showcases the equilibrium bond-length distribution, offering insights into the structural distortions induced in both figures. The 0 K cubic lattice parameter *a* and the elastic constants ($C_{11}$, $C_{12}$, $C_{44}$) are depicted in Fig. 4(c) and (d), respectively. These are plotted against the variance of the bond-length distribution, $\Sigma^{2}$. Mirroring observations from the *Λ* models, the FCC lattice undergoes contraction with escalating bond length disorder. The effect of bond length dispersion at 0 K on the lattice parameter *a* and elastic constants mirrors that of the temperature in a scenario devoid of bond length dispersion. This observation is substantiated by comparing Fig. 4(c) and (d) and Figs. 3(a) and (b). Notably, elastic constants $C_{11}$ and $C_{12}$ diminish as bond disorder rises—a trend consistent with the *Λ*-model outcomes where the elastic stiffness $k_{yy}$ (akin to $C_{11}$) weakens with mounting bond length dispersion. Conversely, the shear elastic constant $C_{44}$ wanes with bond length disorder, albeit at a reduced rate than its counterparts. To put this in context, the value of $k_{xx}$ in the *Λ* model actually escalates with bond length disorder, as illustrated in Fig. S5(c). The decrease in elastic constants as lattice distortion increases aligns with Nöhring’s observation and statement (14) that the elastic constants of true random alloys are consistently lower than those of averaged-atom alloys.

The synergistic impact of temperature and bond length disorder on the lattice parameter and elastic constants is vividly demonstrated in Fig. 4(e)–(h); for the unscaled data, readers can refer to Fig. S6. Utilizing Eq. 8 to define an effective temperature $T_{\mathrm{eff}}$ yields an almost flawless scaling or data collapse, reinforcing the validity of the proposed scaling relation. This lends credence to the idea that thermal and disorder effects in crystalline materials behave in a similar manner to those in the *Λ* models, a hypothesis initially supported by partition function analysis (applicable at least within the regime of low $T_{\mathrm{eff}}$). Both increasing disorder and rising temperature result in lattice contraction and a softening of the elastic constants.

Similar to the *Λ* model, the convergence of random bond lengths and finite-temperature FCC crystal data onto a unified curve underscores the previously observed “equivalence” between temperature and bond length disorder. The value of the parameter *α* in $T_{\mathrm{eff}}$ is determined by fitting it to simulation data at low $T_{\mathrm{eff}}$ for the parameters *a*, $C_{11}$, $C_{12}$, and $C_{44}$; specifically, *α* is found to be 3.6±0.2, 4.5±0.2, 7.0±0.5, and 3.3±0.3, respectively. The distinct values of *α* associated with each quantity stem from the different geometries of the corresponding deformations: dilatation (*a*), uniaxial strain ($C_{11}$), lateral strain ($C_{12}$), and shear ($C_{44}$).

The concept of an effective temperature, denoted as $T_{\mathrm{eff}}$, encapsulates the notion that structural disorder arising from thermal vibrations and local static lattice distortions is statistically indistinguishable. While we illustrate how *T* and *Σ* synergistically combine within the framework of an effective temperature, we have not yet provided an analytical prediction for the coefficients *α*. The value of *α* exhibits a complex dependence on multiple factors, including bond connectivity, lattice geometry, and the direction in which the properties of interest are measured, particularly in the case of tensor properties like $C_{ij}$. As a result, *α* is not a universal constant; it does not possess the same value for all properties within a specific HEA, nor does it remain constant across all HEAs for a given property.

Additionally, we conduct MD simulations on a 3D lattice system characterized by bond strength dispersion while maintaining identical bond lengths. The outcomes of these simulations, as depicted in Fig. S22, reveal that bond strength distribution exerts only a minimal impact on the temperature-dependent behavior of lattice parameters or elastic constants. This outcome is not unexpected, as bond strength dispersion, under conditions of a constant equilibrium bond length, does not induce local lattice distortions at 0 K.

## Discussion and conclusions

The primary objective of this study is to investigate the impact of bond length and atomic size disorder on structural and mechanical properties. The resulting lattice distortion plays a pivotal role in various aspects of compositionally complex and high-entropy alloys, as evidenced by prior research (38–41). To isolate the effects of atomic size variations and the resulting lattice distortions on thermodynamic behavior, we intentionally neglect the influence of bond strength or stiffness variations among alloy components. Our approach is rooted in a statistical mechanics treatment of both distortions arising from thermal fluctuations in bond length and those associated with bond length disorder. We explore these phenomena within a simple 2D model and subsequently validate the emergent features within a 3D crystal lattice using MD simulations.

The primary finding of this study is that the partition function for harmonic bonds, with a single unstretched bond length at a finite temperature (*T*), is equivalent to that of a system where unstretched bond lengths are randomly selected from a Gaussian distribution at T=0 in the 2D (*Λ*) model. This result can be understood by observing that the thermal distribution of bond lengths in a harmonic model follows a Gaussian distribution with a standard deviation proportional to $T^{1/2}$. When the distribution of unstretched bond lengths is also Gaussian with a standard deviation of *Σ*, the two systems exhibit indistinguishable instantaneous configurations. The thermal average bond length and stiffness in a system where unstretched bond lengths are randomly selected are identical to those in a system with a single unstretched bond length at temperature *T*, provided we replace *T* with an effective temperature, denoted as $T_{\mathrm{eff}}=T+\alpha \Sigma^{2}$, where *α* is a constant. These findings have been validated through MD simulations in the 2D system (see Fig. 1).

While a direct analytical evaluation of the partition function for 3D FCC crystals is not feasible, we conduct MD simulations at various temperatures for both scenarios: the single unstretched bond length case and the case where unstretched bond lengths are randomly selected from a Gaussian distribution. Our simulations reveal that the equilibrium cubic lattice parameter (*a*) and the three cubic elastic constants ($C_{11}$, $C_{12}$, and $C_{44}$) for both systems converge onto single curves at an effective temperature ($T_{\mathrm{eff}}$), as illustrated in Figs. 4 and S6. This outcome demonstrates the applicability of the 2D theoretical results to the 3D FCC lattice system. Additionally, similar effective temperature behavior is observed in the FCC model in the presence of the anharmonic term ($k_{3}$). The effect of the strength of anharmonicity is studied for three values of $k_{3}$ and the results are shown in Figs. S11–S13 (representatives for negative thermal expansion, Invar, and positive thermal expansion). Figures S14–S16 show how each of these families of curves collapse onto a single curve each with a different *α* corresponding to each physical quantity *a*, $C_{11}$, $C_{12}$, and $C_{44}$ and for different values of $k_{3}$. We further observe a similar convergence of data for specific properties with the introduction of an effective temperature ($T_{\mathrm{eff}}$) in other lattice structures, as demonstrated in the case of the 3D HCP lattice in Fig. S21.

The finding that temperature and bond length disorder are interchangeable holds intriguing thermodynamic implications for compositionally complex and high-entropy alloys. The contribution of compositional complexity to the system’s entropy is typically calculated using a mean-field or Bragg–Williams approximation based on random site substitution. In an equi-atomic, random solid solution comprising *n* components, the entropy of mixing is represented as $\Delta S_{m}=k_{B}\ln\;n$. This is often regarded as the primary contribution to the change in free energy when transitioning from a homogeneous single-component alloy to a high-entropy alloy, neglecting internal energy effects. Given that the key findings derived from the 2D *Λ* model align well with our MD simulations of the FCC lattice, we revisit the 2D *Λ* model to assess the influence of atomic size and unstretched bond length dispersion on the thermodynamic behavior of our harmonic-bond 3D FCC system.

The partition function of the *Λ* model (see Eq. S9) is $Z=2\pi /\beta \cdot \pi /(\beta_{\mathrm{eff}}bh)=(2\pi^{2}T/bh)(T+\alpha \Sigma^{2})$, where $\beta_{\mathrm{eff}}=1/T_{\mathrm{eff}}$. From this, we derive the Helmholtz free energy (F=−TlnZ), as well as the entropy and internal energy. We can also explicitly add a term corresponding to the noncomposition entropy $\Delta S_{\mathrm{nc}}$ (for an *n* component system) in the free energy


(9) $$F=-T\ln\;\left[nT^{2}\left(1+\frac{\Sigma^{2}}{T}\right)\right]+CT,$$

where *C* is a numerical constant accounting for the detailed geometry of the model. The changes in free energy, entropy and internal energy on going from a mono-dispersed bond length system to one with bond length dispersion are


(10) $$\Delta F=-T\ln\;\left(1+\frac{\Sigma^{2}}{T}\right)\approx -\Sigma^{2}\left(1+\frac{\Sigma^{2}}{2T}\right),$$

(11) $$\Delta S=\ln\;\left(1+\frac{\Sigma^{2}}{T}\right)-{\left(1+\frac{T}{\Sigma^{2}}\right)}^{-1}\approx \frac{1}{2}{\left(\frac{\Sigma^{2}}{T}\right)}^{2},$$

(12) $$\Delta U=-T{\left(1+\frac{\Sigma^{2}}{T}\right)}^{-1}\approx \Sigma^{2}\left(1+\frac{\Sigma^{2}}{T}\right),$$

where we have set α=1 (for simplicity) and the final expressions are the leading order terms in $\Sigma^{2}/T$. As a result, bond length distribution introduces a temperature-dependent entropy component, supplementing the traditional entropy term associated with the random distribution of atoms of different types on lattice sites ($k_{B}\ln\;n$). The contribution of noncompositional entropy ($S_{\mathrm{nc}}$) is also quantified by Nöhring and Curtin (14) using the average-atom EAM potential. Notably, this contribution is relatively small in scale. Their findings regarding the free energy difference (ΔF) align with Eq. 10, particularly in the low-temperature limit.

**Fig. 5.**
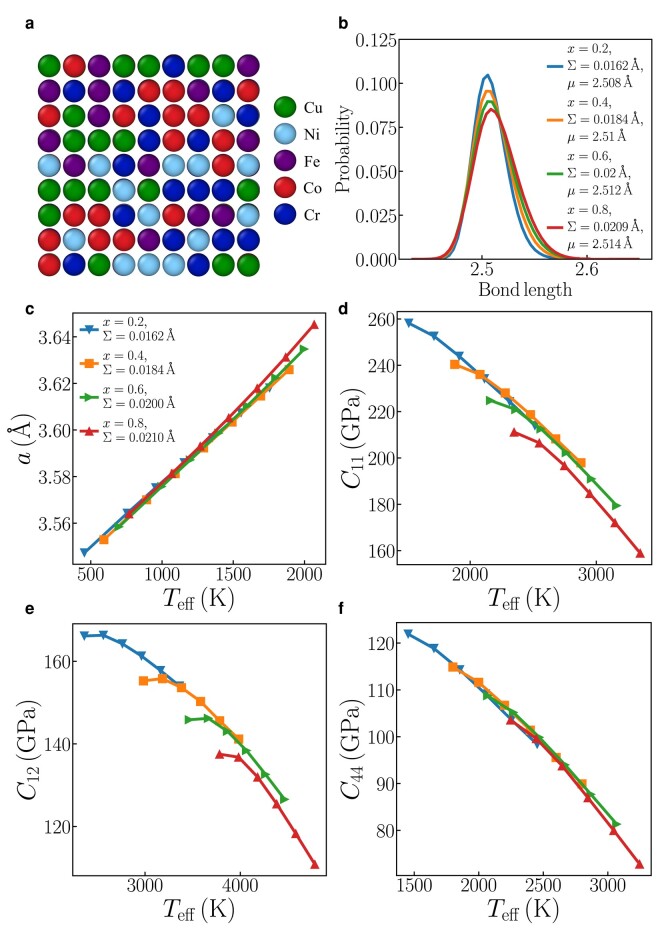
An FCC (CuCr)_*x*_(NiFeCo)_1−*x*_ HEA: a) Relaxed structure (a section of a much larger sample) for x=0.4 and Σ=0.0184 Å. b) Bond-length distribution after relaxation; the legend shows the concentration of CuCr and the corresponding standard deviation *Σ*. c) lattice parameter and (b)–(d) elastic constants ($C_{11}$, $C_{12}$, $C_{44}$) vs. effective temperature for various *Σ* values.

To contextualize the thermodynamic impact of bond length disorder, we have estimated its contribution to several HEAs. Our analysis involves MD simulations for a CuCrNiFeCo alloy system using EAM potentials (42). Figure 5(a) and (b) depict a typical relaxed structure and the associated bond length distribution. These results, as shown in Fig. 5(c)–(f), clearly illustrate that the scaling law involving the effective temperature ($T_{\mathrm{eff}}$) established for simple harmonic systems is also applicable to a real HEA.

We have also calculated the standard deviation of the nearest-neighbor distances for several experimentally realized HEAs. Specifically, for the FCC Cantor alloy (CoCrMnFeNi), we find Σ=0.0303, and for FCC CoCrCuFeNi, Σ=0.0294 (refer to Table S2 for more details). As previously discussed, bond length disorder effectively elevates the temperature above its actual value. At room temperature, this amounts to an increase of 42% for CoCrMnFeNi and 41% for CoCrCuFeNi (details are available in Table S2). At 1,000 K, the influence of bond length disorder is more modest, effectively raising the temperature by 12% for both alloys. It is crucial to highlight that while bond length disorder significantly impacts thermodynamic properties at lower temperatures, its effect on elastic constants and melting temperatures is much less pronounced. Moreover, these impacts are generally subdued in FCC HEAs owing to their lower levels of local lattice disorder (Σ≤5%) (29, 43) (see Figs S18 and S23). When comparing the magnitudes of atomic displacements caused by vibration and bond length effects, it is important to note that in real systems, the intrinsic lattice distortion scale is typically smaller than the vibrational scale. This observation applies to the variations in the atomic radii of the constituent elements, regardless of the presence of fewer or more atom types (cf. Fig. 5(a) and Fig. 6 in Ref. (39) and the Discussion in Ref. (25)).

It is intriguing to observe that one of the classic Hume–Rothery rules posits that stable substitutional solutions exist only when the atomic sizes of the solute and solvent atoms differ by no more than 15% (44, 45). This implies that there could be an upper bound on the extent of atomic size dispersion allowable in HEAs. The relevance of this rule to HEAs is an open question deserving further investigation; for example, see Ref. (46). We should note that in the HEAs we have referenced in Table S2, the maximum value of $\Sigma /\bar{l}$ falls below this 15% threshold.

We demonstrate that temperature and lattice distortion jointly contribute to numerous thermodynamic, structural, and elastic properties via a unified effective temperature, encompassing both the actual temperature and the extent of lattice distortion. Lattice distortion notably enhances the stability of a single-phase solid solution, as commonly found in binary and multicomponent alloys, by elevating the effective temperature. Increasing temperature further reinforces the stability of a solid solution compared to phase separation or ordering. The established scaling law for lattice distortion is validated through simulations in a 2D *Λ* model, a 3D harmonic and anharmonic crystal model, and by employing MD simulations on a real HEA.

## Methods

Molecular dynamics (MD) simulations of the 2D three-atom system depicted in Fig. 1(a) is performed using our in-house MD code, where the masses of the atoms are 1 gm/mole and the velocity Verlet integration time step is taken to be 0.05 fs. Temperature is maintained through a Langevin thermostat with a friction coefficient of 0.2 time units.

3D simulations are performed using the Large-scale Atomic/Molecular Massively Parallel Simulator (LAMMPS) (47). In these simulations, all nearest atoms are connected by elastic harmonic bonds. The equilibrium lattice parameters at finite temperatures are obtained using the Nosé–Hoover thermostat and barostat (*NPT*) with a time step of 1 fs. The elastic constant tensor is obtained by applying strains to the box within *NVT* ensemble. The elastic constants at all temperatures are determined at fixed strains are calculated from $C_{ij}=\Delta \sigma /\varepsilon$, where Δσ represents the stress change and ε is the applied strain. The reported elastic constants are obtained by extrapolating the finite strain results to zero strain at any temperature. Simulations cover a finite temperature range from 300 K to 1,500 K, with intervals of 200 K. The MD results are obtained through time and symmetry averaging in a system comprising 500,000 atoms, following the equilibration process.

The selection of the bond length distribution function, along with the extent of bond length disorder represented by *Σ*, is informed by computational analyses grounded in DFT as well as calculations based on EAM potentials. These analyses cover a wide array of HEAs and are corroborated by multiple studies (29, 43, 48). Empirical observations consistently reveal that the bond length distributions of both the first-nearest neighbor (1NN) and the second-nearest neighbor (2NN) conform to Gaussian patterns. Additionally, the standard deviations of these Gaussian distributions, denoted as *Σ*, have been observed to lie within the interval [0, 0.11]. For a more in-depth comparison between our findings and the DFT/EAM-calculated bond length distributions at 0 K, refer to Figs S18 and S23. Given these empirical and computational insights, our study adopts a Gaussian distribution for bond lengths, setting the standard deviation *Σ* within a slightly extended range of [0, 0.15]. For consistency with real HEA potential cases, Gaussian distributions with the standard deviation between 0 and 0.108 after relaxation at 0 K are used in manuscript. In simulations, we find when standard deviation exceeds 0.2, the structure would be unstable and show continuous shrinkage even at lower finite temperatures.

The core of this study revolves around the face-centered cubic (FCC) lattice, characterized by harmonic springs connecting nearest neighbor atoms. This lattice is simply chosen as an example in this article; the results are applicable to other lattices as well (we discuss nearest-neighbor hexagonal close-packed HCP and second nearest-neighbor bonded body centered cubic BCC, harmonically bonded lattices and see Figs. S19–S21, Table S3).

To provide a broader context, MD simulations are executed on a 3D HEA model employing extant EAM potentials. The objective of these simulations is to elucidate the convergences and divergences between the outcomes generated by our simplified model and those derived from more complex EAM-based calculations.

## Supplementary Material

pgae117_Supplementary_Data

## Data Availability

All data required for main findings of this manuscript are included in the article and supplementary material.
