# Supplementary material for: Scaling laws for lattice distortions: Application to high entropy alloys
Source: PNAS Nexus. 2024 Mar 18;3(4):pgae117. doi: 10.1093/pnasnexus/pgae117 (PMC10983832; doi:10.1093/pnasnexus/pgae117)
Supplement: pgae117_Supplementary_Data [file pgae117_supplementary_data.pdf]

Scaling laws for lattice distortions: application to high entropy alloys

Zhaowei Wang, A. S. L. Subrahmanyam Pattamatta, Jian Han, David J. Srolovitz*

March 13, 2024

1 Reduced quantities

The definition of the reduced quantities employed in the main text and in this Supplementary Material are summarized in Table S1.

Table S1: Reduced quantities. The constants (with dimension) involved are: the unstretched bond length L_0, atomic mass m, bond stiffness K_2 and Boltzmann constant k_B.

Quantity	Quantities with dimension	Reduced quantites	Definition of reduced quantities
Coordinates	X, Y, Z	x, y, z	$\frac{X}{L_0}$, $\frac{Y}{L_0}$, $\frac{Z}{L_0}$
Momenta	P_x, P_y, P_z	p_x, p_y, p_z	$\frac{P_x}{L_0\sqrt{mK_2}}$, $\frac{P_y}{L_0\sqrt{mK_2}}$, $\frac{P_z}{L_0\sqrt{mK_2}}$
Spring length	L	ℓ	$\frac{L}{L_0}$
Temperature	$\mathcal{T}$	T	$\frac{k_\mathrm{B}\mathcal{T}}{K_2L_0^2}$
Inverse temperature	$\mathcal{T}^{-1}$	β	$\frac{K_2L_0^2}{k_\mathrm{B}\mathcal{T}}$
Potential energy of interaction	Φ	ϕ	$\frac{\Phi}{K_2L_0^2}$
Potential energy, kinetic energy	$\mathcal{U}$, $\mathcal{K}$	U, K	$\frac{\mathcal{U}}{K_2L_0^2}$, $\frac{\mathcal{K}}{K_2L_0^2}$
Force	F	f	$\frac{F}{K_2L_0}$
Geometry of Λ-model	H, B	h, b	$\frac{H}{L_0}$, $\frac{B}{L_0}$
Configurational standard deviation	std.	Σ	$\frac{\text{std.}}{L_0}$
Anharmonic spring coefficient	K_3, K_4	k_3, k_4	$\frac{K_3L_0}{K_2}$, $\frac{K_4L_0^2}{K_2}$
Stiffness of Λ-model	K_{xx}, K_{yy}	k_{xx}, k_{yy}	$\frac{K_{xx}}{K_2}$, $\frac{K_{yy}}{K_2}$
Lattice parameter	a	a	$\frac{\mathrm{a}}{L_0}$
Elastic constants	$\mathcal{C}_{ij}$	C_{ij}	$\frac{2\mathcal{C}_{ij}L_0}{K_2}$
Timestep	t	t	$\frac{\mathrm{t}}{\sqrt{m/K_2}}$

2 Harmonic-bond Λ-model

Potential energy landscape

Consider the configuration shown in Fig. 1 of the main text. When the "free" atom is located at $\mathbf{r} = (x, y)$, the total potential energy is

$$U(\mathbf{r}) = \frac{1}{2}\left\{\left[\sqrt{(x+b)^2+(y+h)^2}-1\right]^2+\left[\sqrt{(x-b)^2+(y+h)^2}-1\right]^2\right\}. \tag{S1}$$

The kinetic energy is

$$K(\mathbf{p}) = \frac{1}{2}\left(p_x^2+p_y^2\right), \tag{S2}$$

where $\mathbf{p} = (p_x, p_y)$ is the momentum. The partition function is

$$\mathcal{Z} = \iint e^{-\beta(U+K)}\mathrm{d}\mathbf{r}\mathrm{d}\mathbf{p} = \int e^{-\beta U}\mathrm{d}\mathbf{r}\iint_{-\infty}^{\infty}\exp\left[-\frac{\beta}{2}\left(p_x^2+p_y^2\right)\right]\mathrm{d}p_x\mathrm{d}p_y = \frac{2\pi}{\beta}\int e^{-\beta U}\mathrm{d}\mathbf{r}. \tag{S3}$$

The ensemble average of a quantity A can be calculated by

$$\langle A\rangle = \frac{1}{\mathcal{Z}}\iint Ae^{-\beta(U+K)}\mathrm{d}\mathbf{r}\mathrm{d}\mathbf{p} = \frac{\int Ae^{-\beta U}\mathrm{d}\mathbf{r}}{\int e^{-\beta U}\mathrm{d}\mathbf{r}}. \tag{S4}$$

Since the expression of $U(\mathbf{r})$ is complicated, the integrals in Eq. (S4) cannot be solved analytically. Therefore, we expand Eq. (S1) about $\mathbf{r} = \mathbf{0}$ to 3rd order:

$$U(\mathbf{r}) = b^2x^2 + h^2y^2 - h(2b^2-h^2)x^2y + hb^2y^3 + \mathcal{O}(r^4). \tag{S5}$$

The comparison between the exact potential energy landscape (expressed by Eq. (S1)) and the approximate landscape (Eq. (S5)) is shown in Fig. S1.

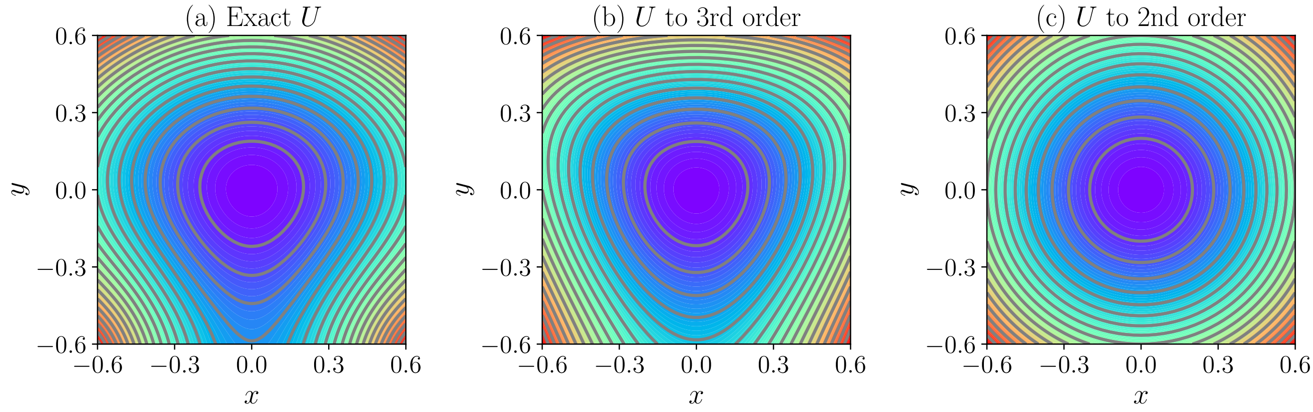

Figure S1: (a) The exact potential energy according to Eq. (S1). (b) The approximate potential energy to the 3rd order according to Eq. (S5). (c) The approximate potential energy to the 2nd order. For this plot, $h = b = 1/\sqrt{2}$.

2.1 Equilibrium position of the "free" atom

We now consider an approximate to the partition function Eq. (S3) in order to analytically determine average properties. Expanding

$$e^{-\beta U} = \exp\left[-\beta\left(b^2x^2 + h^2y^2\right)\right]\exp\left\{\beta\left[h(2b^2 - h^2)x^2y - hb^2y^3\right]\right\}, \tag{S6}$$

about $\mathbf{r} = \mathbf{0}$ yields:

$$\exp\left\{\beta\left[h(2b^2 - h^2)x^2y - hb^2y^3\right]\right\} = 1 + \beta\left[h(2b^2 - h^2)x^2y - hb^2y^3\right] + \mathcal{O}(r^6). \tag{S7}$$

Thus,

$$e^{-\beta U} \approx \exp\left[-\beta\left(b^2x^2 + h^2y^2\right)\right]\left\{1 + \beta\left[h(2b^2 - h^2)x^2y - hb^2y^3\right]\right\}. \tag{S8}$$

Then, the approximate partition function is

$$\mathcal{Z} \approx \int_{-\infty}^{\infty}\int_{-\infty}^{\infty}\exp\left[-\beta\left(b^2x^2 + h^2y^2\right)\right]\left\{1 + \beta\left[h(2b^2 - h^2)x^2y - hb^2y^3\right]\right\}\mathrm{d}x\mathrm{d}y = \frac{\pi}{\beta bh}. \tag{S9}$$

The first moments are

$$\int xe^{-\beta U}\mathrm{d}\mathbf{r} \approx \int_{-\infty}^{\infty}\int_{-\infty}^{\infty}\exp\left[-\beta\left(b^2x^2 + h^2y^2\right)\right]\left\{x + \beta\left[h(2b^2 - h^2)x^3y - hb^2xy^3\right]\right\}\mathrm{d}x\mathrm{d}y = 0, \tag{S10}$$

$$\begin{aligned}\int ye^{-\beta U}\mathrm{d}\mathbf{r} &\approx \int_{-\infty}^{\infty}\int_{-\infty}^{\infty}\exp\left[-\beta\left(b^2x^2 + h^2y^2\right)\right]\left\{y + \beta\left[h(2b^2 - h^2)x^2y^2 - hb^2y^4\right]\right\}\mathrm{d}x\mathrm{d}y \\ &= -\frac{\pi}{4\beta^2b^3h^4}\left(3b^4 + h^4 - 2b^2h^2\right).\end{aligned} \tag{S11}$$

So, the equilibrium position at temperature β is

$$\langle x\rangle^0 = 0, \quad \langle y\rangle^0 = -\frac{3b^4 + h^4 - 2b^2h^2}{4\beta b^2h^3}. \tag{S12}$$

2.2 Stiffness of the Λ-model

We now derive the temperature dependence of the stiffness of the Λ-model. We only focus on the motion of vertex atom along the y-direction in response to a force along the y-direction. Equation (S1) with $x = 0$ is

$$U(y) = \left[\sqrt{b^2 + (y+h)^2} - 1\right]^2. \tag{S13}$$

In order to show the temperature dependence of stiffness, we expand U about $y = 0$ to the 4th order:

$$U = h^2y^2 + hb^2y^3 + \frac{1}{4}b^2(b^2 - 4h^2)y^4 + \mathcal{O}(y^5). \tag{S14}$$

Applying a force f in the y-direction yields the enthalpy $U - fy$. The inverse stiffness is

$$k_y^{-1} = \left.\frac{\partial\langle y\rangle^0}{\partial f}\right|_{f=0}. \tag{S15}$$

This is valid in the limit that the applied force $\to 0$. The equilibrium position and its variation with the applied force are

$$\langle y\rangle^0 = \frac{\int y e^{-\beta(U-fy)}\mathrm{d}y}{\int e^{-\beta(U-fy)}\mathrm{d}y} \quad \Rightarrow \quad \frac{\partial\langle y\rangle^0}{\partial f} = \frac{\beta\int y^2 e^{-\beta(U-fy)}\mathrm{d}y}{\int e^{-\beta(U-fy)}\mathrm{d}y} - \beta\left[\frac{\int y e^{-\beta(U-fy)}\mathrm{d}y}{\int e^{-\beta(U-fy)}\mathrm{d}y}\right]^2. \tag{S16}$$

In the $f \to 0$ limit, this yields

$$k_y^{-1} = \left.\frac{\partial\langle y\rangle^0}{\partial f}\right|_{f=0} = \beta\left[\langle y^2\rangle^0 - \left(\langle y\rangle^0\right)^2\right]. \tag{S17}$$

This shows that the inverse stiffness is determined by the standard deviation of y. β decreases as T increases while the standard deviation increases with T. So, to show the temperature dependence, we examine the standard deviation at higher order: this is why we expanded U to the 4th order in Eq. (S14).

We derive the standard deviation of the vertex atom position based upon Eq. (S14). The partition function is $\mathcal{Z} = \int e^{-\beta U}\mathrm{d}y$, where the integrand is

$$e^{-\beta U} = e^{-\beta h^2 y^2}\left\{1 - \beta\left[hb^2y^3 + \frac{1}{4}b^2(b^2-4h^2)y^4\right] + \mathcal{O}(y^5)\right\}. \tag{S18}$$

With this approximation,

$$\mathcal{Z} = \int_{-\infty}^{\infty} e^{-\beta h^2 y^2}\left\{1 - \beta\left[hb^2y^3 + \frac{1}{4}b^2(b^2-4h^2)y^4\right] + \mathcal{O}(y^5)\right\}\mathrm{d}y = \frac{1}{h}\sqrt{\frac{\pi}{\beta}}\left[1 - \frac{b^2}{h^2}\left(\frac{b^2}{4h^2} - 1\right)\frac{3}{4\beta}\right]. \tag{S19}$$

The first moment is

$$\int y e^{-\beta U}\mathrm{d}y = \int_{-\infty}^{\infty} e^{-\beta h^2 y^2}\left\{y - \beta\left[hb^2y^4 + \frac{1}{4}b^2(b^2-4h^2)y^5\right] + \mathcal{O}(y^6)\right\}\mathrm{d}y \approx -\frac{3\sqrt{\pi}}{4\beta^{3/2}}\frac{b^2}{h^4}. \tag{S20}$$

The second moment is

$$\begin{aligned}\int y^2 e^{-\beta U}\mathrm{d}y &= \int_{-\infty}^{\infty} e^{-\beta h^2 y^2}\left\{y^2 - \beta\left[hb^2y^5 + \frac{1}{4}b^2(b^2-4h^2)y^6\right] + \mathcal{O}(y^7)\right\}\mathrm{d}y \\ &\approx \frac{\sqrt{\pi}}{2\beta^{3/2}h^3} - b^2(b^2-4h^2)\frac{15\sqrt{\pi}}{32\beta^{5/2}h^7}.\end{aligned} \tag{S21}$$

The average position of the vertex atom is

$$\langle y\rangle^0 = \frac{1}{\mathcal{Z}}\int y e^{-\beta U}\mathrm{d}y = \frac{-12hb^2}{16h^4\beta - 3b^2(b^2-4h^2)}. \tag{S22}$$

A quantity which might be interesting for us is $\tan(\gamma_0/2) = b/h \equiv \hat{b}$. So, we reorganize the above equation to

$$h\langle y\rangle^0 = \frac{-12\hat{b}^2}{16\beta - 3\hat{b}^2(\hat{b}^2-4)}. \tag{S23}$$

The mean square position of the vertex atom is

$$\langle y^2\rangle^0 = \frac{1}{\mathcal{Z}}\int y^2 e^{-\beta U}\mathrm{d}y = \frac{16h^4\beta - 15b^2(b^2-4h^2)}{2h^2\beta\left[16h^4\beta - 3b^2(b^2-4h^2)\right]}. \tag{S24}$$

Similarly, we can express it by $\hat{b}$:

$$h^2\langle y^2\rangle^0 = \frac{16\beta - 15\hat{b}^2(\hat{b}^2-4)}{2\beta\left[16\beta - 3\hat{b}^2(\hat{b}^2-4)\right]}. \tag{S25}$$

Thus,

$$k_y^{-1} = \left.\frac{\partial\langle y\rangle^0}{\partial f}\right|_{f=0} = \frac{\beta}{h^2}\left[h^2\langle y^2\rangle^0 - \left(h\langle y\rangle^0\right)^2\right] = \frac{1}{h^2}\frac{128\beta^2 - 288\hat{b}^2(\hat{b}^2-2)\beta + 22.5\hat{b}^4(\hat{b}^2-4)^2}{\left[16\beta - 3\hat{b}^2(\hat{b}^2-4)\right]^2}. \tag{S26}$$

The stiffness, expressed as a function of temperature, is

$$k_y(T) = \frac{h^2\left[16 - 3\hat{b}^2(\hat{b}^2-4)T\right]^2}{128 - 288\hat{b}^2(\hat{b}^2-2)T + 22.5\hat{b}^4(\hat{b}^2-4)^2T^2}. \tag{S27}$$

We can see that $k_y(T=0) = 2h^2$. When $\gamma_0 = 0$, the Λ-model reduces to a single spring and the stiffness of a single bond does not change with temperature.

We can understand the negative temperature dependence of the stiffness based on the curvature of the potential energy, as shown in Fig. S2. As temperature increases, $\langle y\rangle^0$ decreases and, according to Fig. S2b, the curvature decreases. Lower curvature in the potential energy landscape at the mean position of the vertex atom implies lower stiffness.

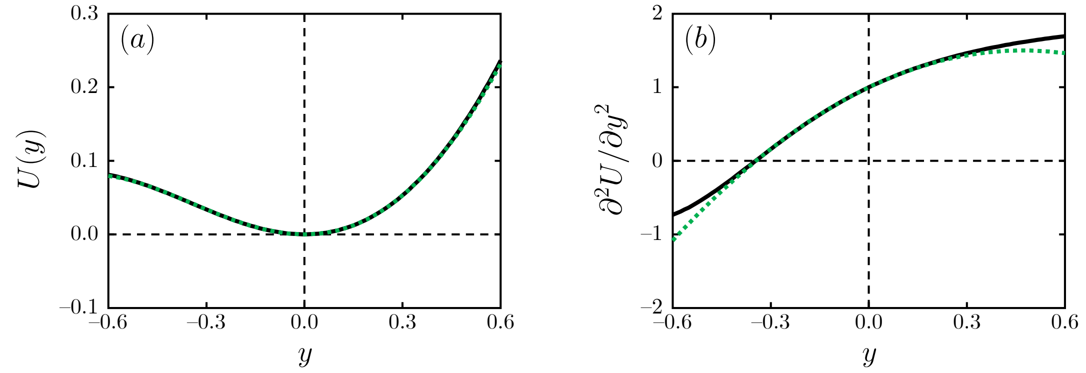

Figure S2: (a) The exact potential energy, Eq. (S13) (black solid line), and the approximate potential energy, Eq. (S14) (green dotted line). For this plot, $h = b = 1/\sqrt{2}$. (b) The curvatures of two lines in (a).

2.3 Simulation results

The molecular dynamics (MD) simulation results for the harmonic-bond Λ-model are shown as the solid lines in Figs. S3a and b. The corresponding theoretical predictions are shown as the dashed lines in the same figures.

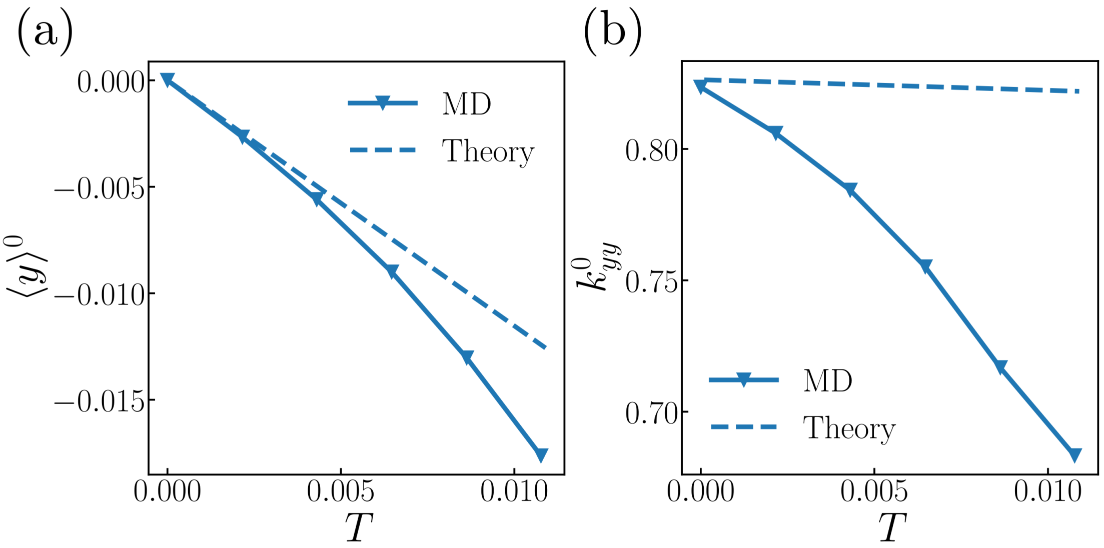

Figure S3: Comparison between analytic (dashed lines) and simulated results (solid lines) for (a) $\langle y \rangle^0$ and (b) k_{yy}^0 vs. temperature based on a harmonic-bond Λ-model.

The molecular dynamics (MD) simulation results for the harmonic-bond FCC model are shown in Fig. S4.

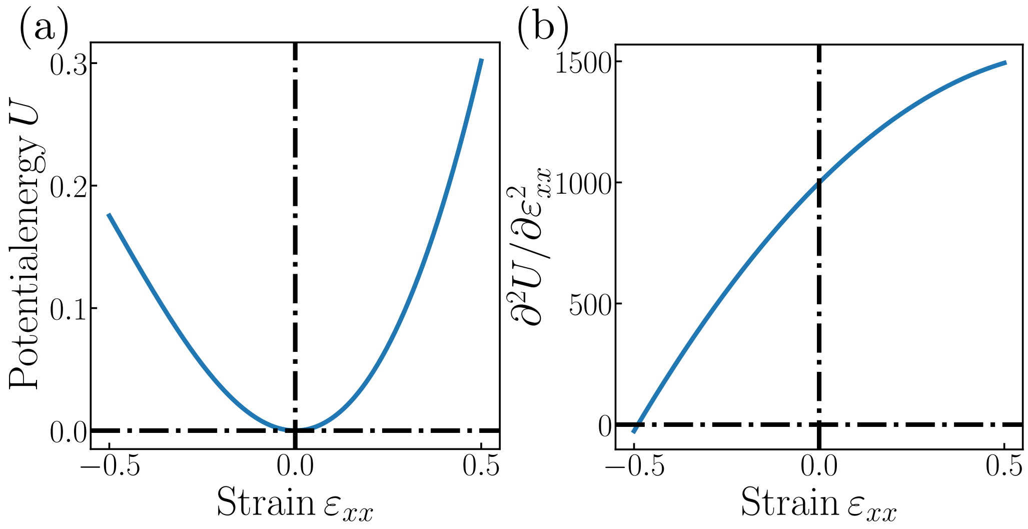

Figure S4: Simulated results of (a) potential energy per atom vs. applied normal strain, (b) second derivative of potential energy with respect to strain based on a nearest-neighbour harmonic-bond FCC model.

3 An ensemble of random harmonic-bond Λ-models

3.1 Effective temperature

We first focus on the effect of a dispersion in bond length at 0 K in the Λ-model where the equilibrium spring lengths of the two springs are randomly assigned according to the normal distribution about 1 with standard deviation Σ:

$$\mathcal{P}(\ell_0) = \frac{1}{\Sigma\sqrt{2\pi}} \exp\left[-\frac{(\ell_0-1)^2}{2\Sigma^2}\right]. \tag{S28}$$

A vertex atom located at $\mathbf{r}$ corresponds to particular bond lengths ℓ_1 and ℓ_2, i.e., $x = x(\ell_1, \ell_2)$ and $y = y(\ell_1, \ell_2)$, or $\ell_1 = \ell_1(x, y)$ and $\ell_2 = \ell_2(x, y)$. The probability that the atom is located at $\mathbf{r}$ is

$$\mathcal{P}(\mathbf{r}) = \mathcal{P}(\ell_1(\mathbf{r}))\mathcal{P}(\ell_2(\mathbf{r})), \tag{S29}$$

where $\mathcal{P}(\ell_1)$ and $\mathcal{P}(\ell_2)$ follow Eq. (S28). An effective potential energy $U_{\text{eff}}(\mathbf{r})$ can be defined such that

$$\mathcal{P}(\mathbf{r}) \equiv \frac{e^{-\beta U_{\text{eff}}(\mathbf{r})}}{\mathcal{Z}_{\text{eff}}}. \tag{S30}$$

Hence,

$$U_{\text{eff}}(\mathbf{r}) = -\beta^{-1}\left[\ln\mathcal{P}(\ell_1) + \ln\mathcal{P}(\ell_2) + \ln\mathcal{Z}_{\text{eff}}\right] = \frac{1}{2\beta\Sigma^2}\left[(\ell_1-1)^2 + (\ell_2-1)^2\right] + \frac{1}{\beta}\ln\left(\frac{2\pi\Sigma^2}{\mathcal{Z}_{\text{eff}}}\right).$$

According to the Λ-model geometry,

$$\ell_1 = \sqrt{(x+b)^2 + (y+h)^2}, \quad \ell_2 = \sqrt{(x-b)^2 + (y+h)^2}. \tag{S31}$$

So, Eq. (S31) becomes

$$U_{\text{eff}}(\mathbf{r}) = \frac{1}{2\beta\Sigma^2}\left\{\left[\sqrt{(x+b)^2+(y+h)^2}-1\right]^2 + \left[\sqrt{(x-b)^2+(y+h)^2}-1\right]^2\right\} + \frac{1}{\beta}\ln\left(\frac{2\pi\Sigma^2}{\mathcal{Z}_{\text{eff}}}\right). \tag{S32}$$

In comparison with Eq. (S1), we find that if

$$\frac{1}{2} = \frac{1}{2\beta\Sigma^2} \quad \Rightarrow \quad \Sigma^2 = \beta^{-1} = T, \tag{S33}$$

$U_{\text{eff}}(\mathbf{r})$ differs from $U(\mathbf{r})$ only by a constant. They are exactly same only when $\mathcal{Z}_{\text{eff}} = 2\pi\Sigma^2$; however, this is not guaranteed. Since $U_{\text{eff}}(\mathbf{r})$ differs from $U(\mathbf{r})$ by a constant at any temperature, the equilibrium positions and standard deviations resulted from $U_{\text{eff}}(\mathbf{r})$ and $U(\mathbf{r})$ are the same. Equation (S33) indicates the equivalence between Σ^2 and T. So, when both the bond length randomness and thermal vibrations are present, we can simply consider a system without bond length randomness but at an effective temperature:

$$T_{\text{eff}} = T + \Sigma^2. \tag{S34}$$

3.2 Simulation results

Figures S5a and b show the MD results for an ensemble of random harmonic-bond Λ-models. We find that neither $\langle y \rangle$ nor k_{yy} can be well scaled by $T + \Sigma^2$. Figure S5c shows the MD results for temperature dependence of stiffness k_{xx}. We find that k_{xx} increases with the increase of either temperature T or bond length disorder Σ^2.

In the main text, we suggested to define the effective temperature as $T_{\text{eff}} = T + \alpha\Sigma^2$. The results of including the geometric coefficient α in is shown in the main text.

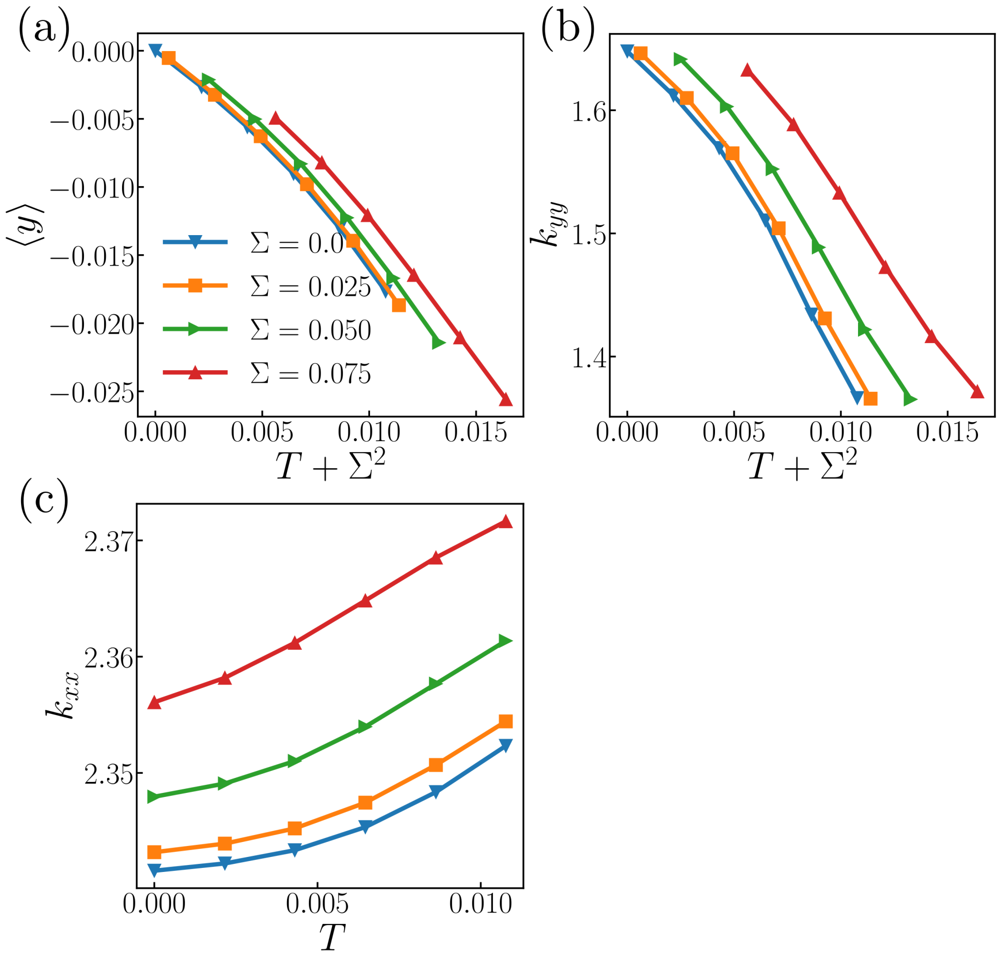

Figure S5: MD results for an ensemble of random harmonic-bond Λ-models.(a) Ensemble-average equilibrium position $\langle y \rangle$ and (b) stiffness k_{yy} vs. $T+\Sigma^2$.(c) Temperature dependence of stiffness k_{xx}.

4 Random, harmonic-bond FCC crystal

The data for the MD simulations of random, harmonic-bond FCC crystal are shown in Fig. S6.

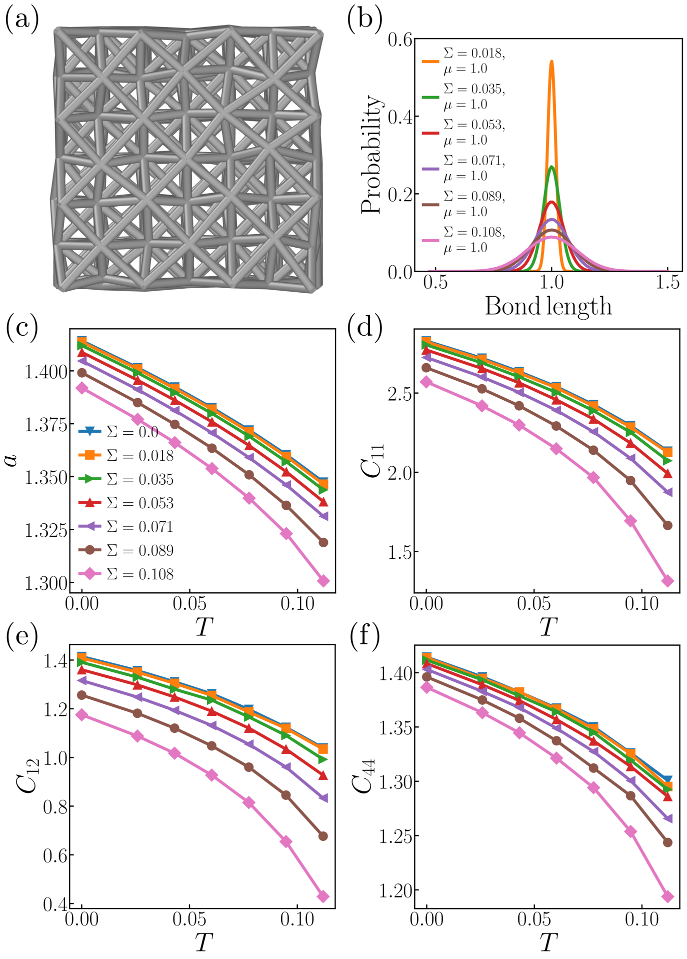

Figure S6: MD results for random, harmonic-bond FCC crystal. (a) The lattice parameter vs. temperature with various randomness Σ. (b), (c) and (d): The cubic elastic constants vs. temperature with various randomness Σ, sharing same legend in (a).

The data for MD simulations of random, harmonic-bond (including second-nearest neighbors) FCC crystal are shown in Fig. S7.

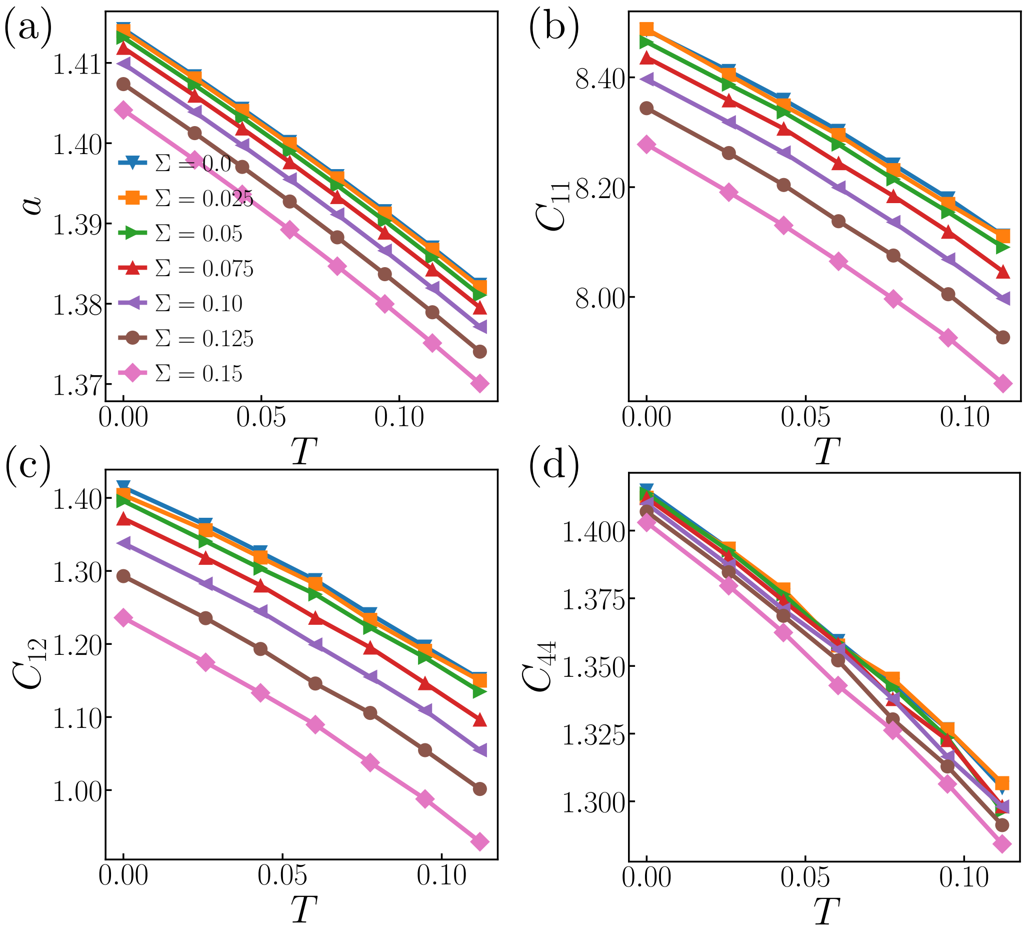

Figure S7: MD results for random, harmonic-bond FCC crystal (second-nearest neighbors are connected by harmonic bonds, sharing the same stiffness as nearest harmonic bonds). (a) The lattice parameter vs. temperature with various randomness Σ. (b), (c) and (d): The cubic elastic constants vs. temperature with various randomness Σ, sharing same legend in (a).

5 Anharmonic Effects

Both the Λ model and the FCC lattice experience contraction when subjected to increased heat or augmented bond length disorder. However, the majority of real crystalline systems demonstrate expansion when exposed to heat. This expansion phenomenon is well-knownly linked to bond anharmonicity, where bonds exhibit greater rigidity under compression than tension. The balancing act between these opposing influences can yield materials whose densities or dimensions remain constant during heating, known as the Invar effect.

Typically, the increase in lattice parameters during heating coincides with a decrease in elastic constants, primarily because bonds become less rigid at larger lattice parameters—reflecting the same anharmonicity responsible for positive coefficients of thermal expansion. Consequently, the effects of bond anharmonicity are expected to alter the impacts of temperature and bond disorder in the harmonic-bond model. While the ideal scenario might entail a material with temperature-independent elastic constants, known as the Elinvar effect, this remains unrealistic in this system. This is due to the fact that in the harmonic-bond model, heating (along with bond disorder) results in diminishing elastic constants, and anharmonic bonds further reinforce this trend.

To incorporate bond anisotropy, we modify the harmonic bond potential described in Eq. (S35). This modification involves considering an arbitrary pairwise potential for bonds, which can be expanded as follows:

$$\phi = \frac{1}{2!}\left(\ell - 1\right)^2 + \frac{1}{3!}k_3\left(\ell - 1\right)^3 + \frac{1}{4!}k_4\left(\ell - 1\right)^4 + \dots, \tag{S35}$$

where the coefficients k_m are specifically selected to regulate the degree of anharmonicity present in the system. While the inclusion of the 3rd order term is adequate to incorporate anharmonicity effects, retaining an even higher order term becomes necessary to ensure that the bond length remains bounded at finite temperatures, such as in MD simulations.

5.1 2D: an anharmonic-bond Λ-model

We consider the same Λ-model as above, but now with the two anharmonic bonds of equilibrium length 1. When a bond length changes to ℓ, the potential energy of this bond is

$$\phi(\ell) = \frac{1}{2}(\ell - 1)^2 + \frac{1}{6}k_3(\ell - 1)^3. \tag{S36}$$

Note that usually $k_3 < 0$. This is schematically plotted in Fig. S8.

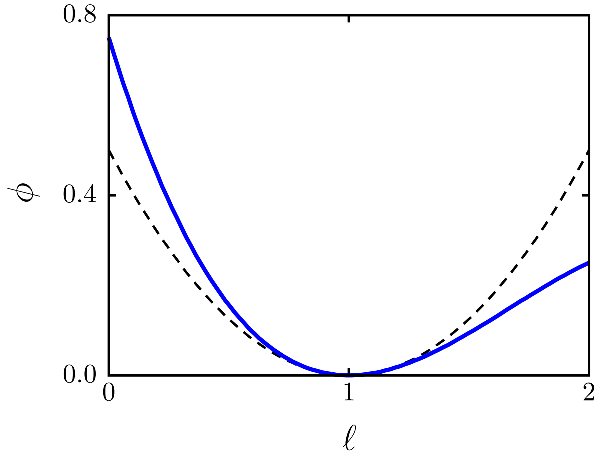

Figure S8: Potential energy of an anharmonic bond, i.e., the plot of Eq. (S36) with $k_3 = -1.5$. The dashed line is the energy with the 3rd order term neglected.

The total potential energy of the Λ-model with anharmonic bonds is

$$U_{\mathrm{A}}(\mathbf{r}) = U(\mathbf{r}) + \frac{1}{6}k_3\left\{\left[\sqrt{(x+b)^2 + (y+h)^2} - 1\right]^3 + \left[\sqrt{(x-b)^2 + (y+h)^2} - 1\right]^3\right\}, \tag{S37}$$

where $U(\mathbf{r})$ is the expression of Eq. (S1). For convenience, define a function:

$$U_3(\mathbf{r}) \equiv \left[\sqrt{(x+b)^2 + (y+h)^2} - 1\right]^3 + \left[\sqrt{(x-b)^2 + (y+h)^2} - 1\right]^3. \tag{S38}$$

By Taylor expansion about $\mathbf{r} = \mathbf{0}$ to the 3rd order,

$$U_3(\mathbf{r}) = 6hb^2x^2y + 2h^3y^3 + \mathcal{O}(r^4). \tag{S39}$$

Substituting Eqs. (S5) and (S39) into Eq. (S37), we find

$$U_{\mathrm{A}}(\mathbf{r}) = b^2x^2 + h^2y^2 - h\left[(2-k_3)b^2 - h^2\right]x^2y + h\left(b^2 + \frac{1}{3}k_3h^2\right)y^3 + \mathcal{O}(r^4). \tag{S40}$$

First, it is interesting to note that there is a particular geometry and k_3 value such that the anharmonic system becomes almost harmonic in the neighbor of $\mathbf{r} = \mathbf{0}$. The 3rd order terms in Eq. (S40) are zero when

$$b = \frac{1}{2}, \quad h = \frac{\sqrt{3}}{2}, \quad k_3 = -1. \tag{S41}$$

This means that the Λ model bond angle $\gamma_0 = 60°$; i.e., the geometry seen in Fig. 1 of the main text becomes an equilateral triangle. Based on this geometry and bond parameter (Eq. (S41)), the comparison between the exact potential Eq. (S37) and the approximate potential Eq. (S40) is shown in Fig. S9. We see that under the condition ofEq. (S41) the energy landscape is nearly elliptical in the vicinity of the minimum.

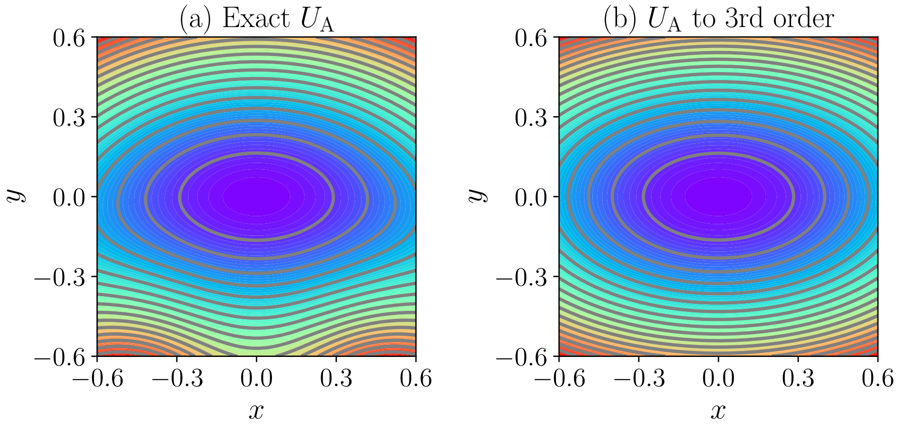

Figure S9: (a) The exact potential energy according to Eq. (S37). (b) The approximate potential energy to the 3rd order according to Eq. (S40). For this plot, the geometry and spring parameter are set by Eq. (S41).

Second, we consider how to achieve an Invar Λ model (i.e., one in which the vertex atom position is temperature-independent). First, we derive $\langle y\rangle^0$. The partition function is

$$\mathcal{Z} \approx \frac{\pi}{\beta bh}. \tag{S42}$$

The first moments are

$$\int xe^{-\beta U_{\mathrm{A}}}\mathrm{d}\mathbf{r} \approx 0, \tag{S43}$$

$$\int ye^{-\beta U_{\mathrm{A}}}\mathrm{d}\mathbf{r} \approx \int_{-\infty}^{\infty}\int_{-\infty}^{\infty} \exp\left[-\beta\left(b^2x^2 + h^2y^2\right)\right]\left\{y + \beta\left[h\left((2-k_3)b^2 - h^2\right)x^2y^2 - h\left(b^2 + \frac{1}{3}k_3h^2\right)y^4\right]\right\}\mathrm{d}x[illegible]$$
$$= -\frac{\pi}{4\beta^2b^3h^4}\left[3b^4 + h^4 - 2(1-k_3)b^2h^2\right]. \tag{S44}$$

So, the average coordinates are

$$\langle x\rangle^0 = 0, \quad \langle y\rangle^0 = -\frac{1}{4\beta b^2h^3}\left[3b^4 + h^4 - 2(1-k_3)b^2h^2\right]. \tag{S45}$$

Again, we can express $\langle y \rangle^0$ by $\hat{b} \equiv \tan(\gamma_0/2)$:

$$h\langle y \rangle^0 = -\frac{1}{4\beta h}\left[3\hat{b}^2 + \frac{1}{\hat{b}^2} - 2(1-k_3)\right]. \tag{S46}$$

The Invar effect is achieved when $\langle y \rangle^0 = 0$ independent of T, which is possible only when $k_3 \leq 1-\sqrt{3}$. Assuming that $k_3 \leq 1-\sqrt{3}$ is satified, the Invar condition is satisfied when

$$\tan^2(\gamma_0/2) = \hat{b}^2 = \frac{1-k_3 \pm \sqrt{(1-k_3)^2-3}}{3}. \tag{S47}$$

There are two roots γ_1 and γ_2. When $\gamma_1 < \gamma_0 < \gamma_2$, Λ expands as T increases; otherwise, Λ shrinks as T increases; see Fig. S10. We find that when the bonds are anharmonic, we can obtain thermal expansion, thermal contraction or Invar by tuning the Λ geometry (i.e., the bond angle γ_0) and k_3.

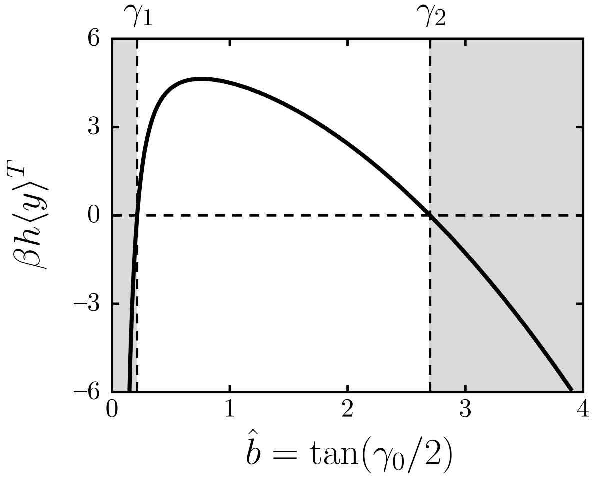

Figure S10: Plot of Eq. (S46) with $k_3 = -10$. Two roots correspond to the bond angles γ_1 and γ_2, with which Invar is achieved. When $\gamma_1 < \gamma_0 < \gamma_2$, Λ expands as T increases; otherwise, Λ shrinks as T increases.

5.2 3D: Random, anharmonic-bond FCC crystal

We conduct similar MD simulations on the anharmonic-bond FCC crystal, exploring its behavior concerning temperature variations, bond length disorder, and the coefficients governing bond anharmonicity. The bond anharmonicity is described using Eq. (S35), truncated at the 4th order. For the bond energy to exhibit a single minimum, the coefficients must adhere to $\frac{8}{3}\kappa < k_3 < 0$, where $\kappa = k_4/k_3$ (ensure that the non-linear model could cover the linear spring model, i.e., when $k_3 \to 0$, the whole bond can devolve to a harmonic spring bond). Our focus centers on the specific scenario of $k_3 = -4.5$ and $\kappa = -2$ (implying $k_4 = 9$). For information on all lattice parameters and elastic constants with various anharmonicities, please refer to Figs. S11, S12 and S13.

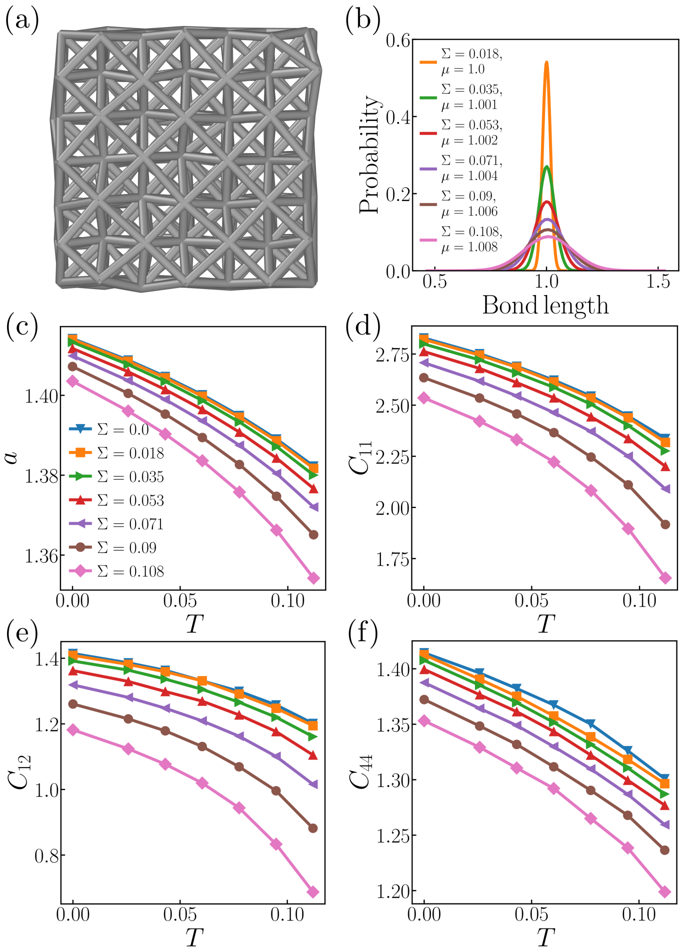

Figure S11: MD results for anharmonic-bond FCC crystal with $k_3 = -1.5$. (a) The lattice parameter vs. temperature with various randomness Σ. (b), (c) and (d): The cubic elastic constants vs. temperature with various randomness Σ, sharing same legend in (a).

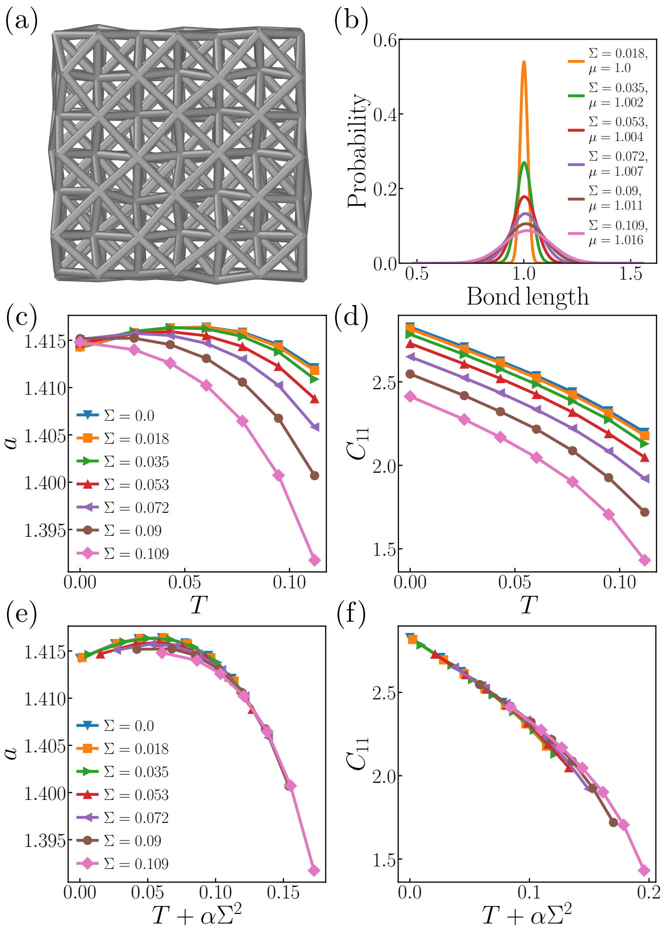

Figure S12: MD results for anharmonic-bond FCC crystal with $k_3 = -3$. (a) The lattice parameter vs. temperature with various randomness Σ. (b), (c) and (d): The cubic elastic constants vs. temperature with various randomness Σ, sharing same legend in (a).

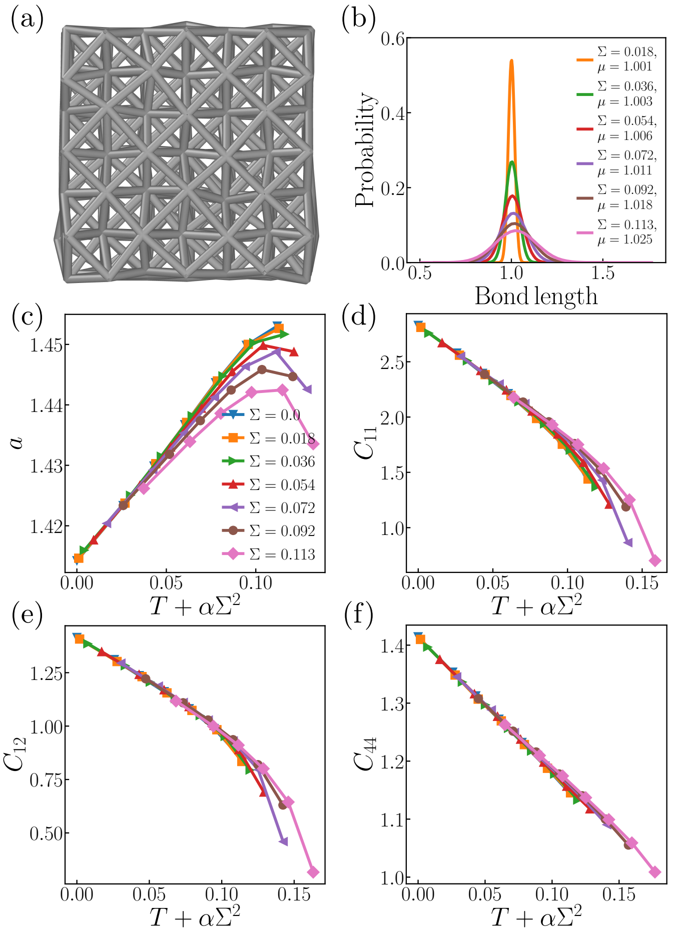

Figure S13: MD results for anharmonic-bond FCC crystal with $k_3 = -4.5$. (a) The lattice parameter vs. temperature with various randomness Σ. (b), (c) and (d): The cubic elastic constants vs. temperature with various randomness Σ, sharing same legend in (a).

After examining the lattice parameter data, it becomes evident that anharmonicity can induce thermal expansion at low temperatures and thermal contraction at high temperatures. This suggests that the geometric effect responsible for thermal contraction in the harmonic model prevails over bond anharmonicity at elevated temperatures, while the inverse holds true for lower temperatures. In real material systems, it is plausible that the material might undergo melting before lattice contraction due to geometrical anharmonicity becomes prominent. As previously discussed, the influence of bond anharmonicity on the elastic constants does not fundamentally alter the general trend of elastic constants decreasing with rising temperatures and increasing bond length disorder.

Fig. S12(c) shows that "near" zero thermal expansion/contraction (Invar effect) is possible in the anharmonic-bond, bond length disordered system over a finite temperature range; e.g., see the case of $\Sigma = 0.035$ for $0 \leq T < 0.05$. On the other hand, an Elinvar effect is not observed for any range of T for any degree of bond disorder (see Fig. S12(d)).

Regarding the disordered FCC crystal with harmonic bonds, the data pertaining to bond disorder exhibit a unified behavior, collapsing onto a singular curve when plotted against $T_{\text{eff}} = T + \alpha\Sigma^2$. This observation holds true for both the lattice parameter and elastic constant data, as shown in Figs. S14-S16. Additionally, from Fig. S17 it suggests that, fundamentally, all three elastic constants can share a common scaling coefficient in this kind of anharmonic crystals. This demonstration underscores the applicability of the near equivalence or scaling of temperature and bond disorder effects, initially observed in the harmonic-bond model, to materials exhibiting anharmonicity.

The (unscaled) data from the MD simulations of random, anharmonic-bond FCC crystals are shown in Fig. S11 for $k_3 = -1.5$, Fig. S12 for $k_3 = -3$, and Fig. S13 for $k_3 = -4.5$. The same data are replotted with respect to the effective temperature $T_{\text{eff}} = T + \alpha\Sigma^2$ in Figs. (S14)-(S16) using values of α obtained by fitting at low T_{eff}.

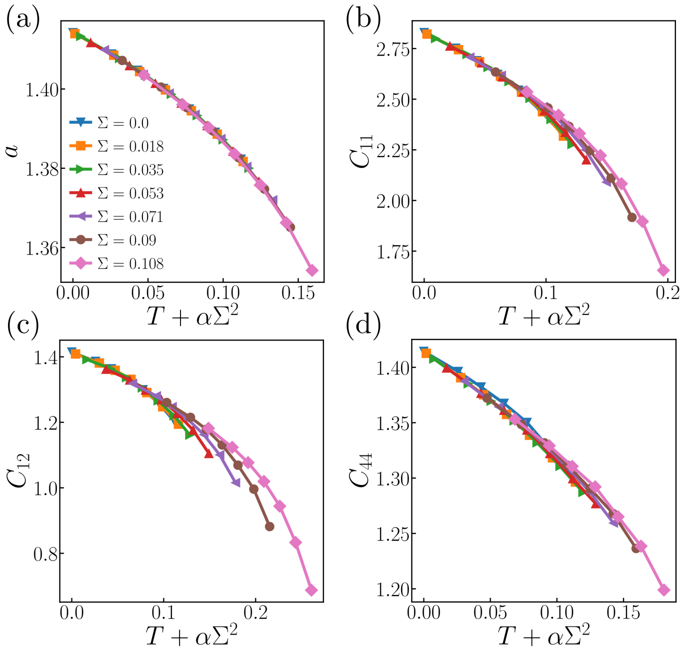

Figure S14: Replot of Fig. S11 with the horizontal axes changed to $T + \alpha\Sigma^2$. The best fit values of α are 2.10±0.03, 3.75±0.2, 6.61±0.16, and 3.04±0.07 for a, C_{11}, C_{12}, and C_{44}, respectively.

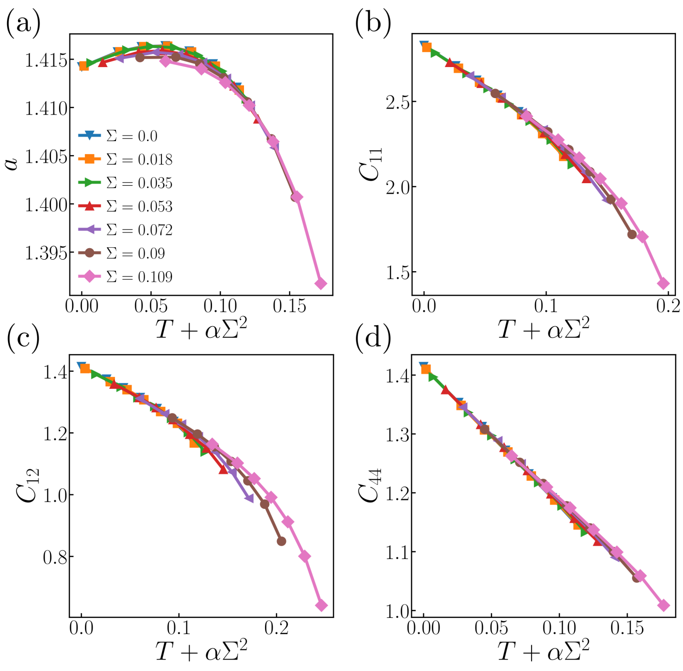

Figure S15: Replot of Fig. S12 with the horizontal axes changed to $T + \alpha\Sigma^2$. The best fit values of α are 2.69±0.2, 3.73±0.05, 5.96±0.5, and 2.88±0.03 for a, C_{11}, C_{12}, and C_{44}, respectively.

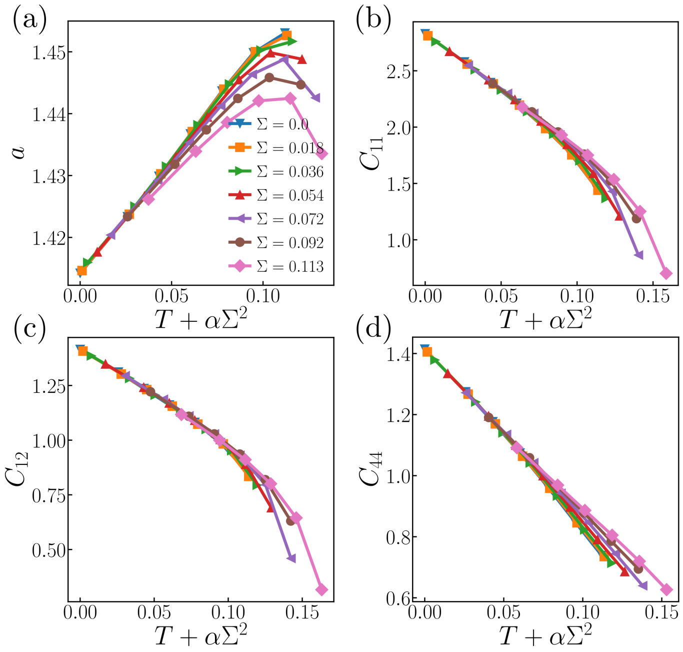

Figure S16: Replot of Fig. S13 with the horizontal axes changed to $T + \alpha\Sigma^2$. The best fit values of α are 1.66±0.17, 2.84±0.05, 3.04±0.05, and 2.59±0.06 for a, C_{11}, C_{12}, and C_{44}, respectively.

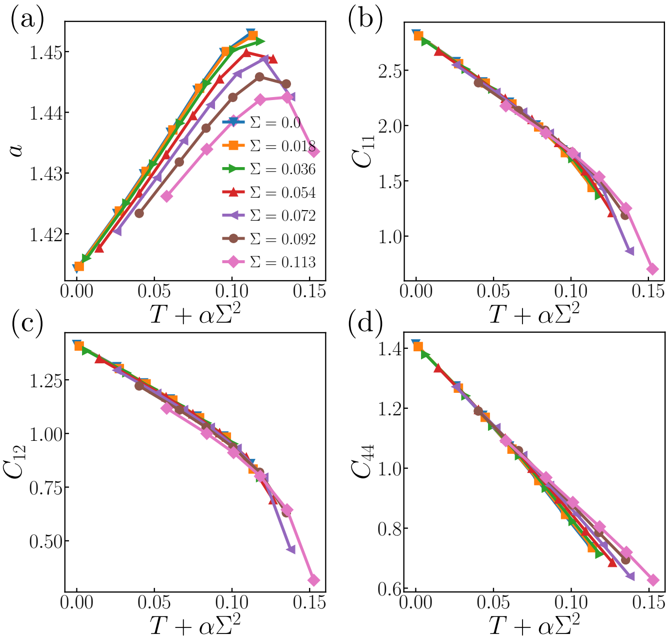

Figure S17: Replot of Fig. S13 with the horizontal axes changed to $T + \alpha\Sigma^2$ with $k_3 = -4.5$ and $k_4 = 9.0$. Here the scaling coeffcient is set to be constant as 2.575.

6 Data for Specific HEAs

HEAs	Structure	a (Å)	$\bar{l}$ (Å)	Σ ($\times 10^{-2}$ Å)	$\Sigma/\bar{l}$	B (GPa)	$K_2 = 3B\bar{l}/2\sqrt{2}$ (Pa·m)	$T_{300\mathrm{K}}$ ($\times 10^{-3}$)	$T_{600\mathrm{K}}$ ($\times 10^{-3}$)	$T_{1000\mathrm{K}}$ ($\times 10^{-3}$)	$\frac{(\Sigma/\bar{l})^2}{T_{300\mathrm{K}}}$	$\frac{(\Sigma/\bar{l})^2}{T_{600\mathrm{K}}}$	$\frac{(\Sigma/\bar{l})^2}{T_{1000\mathrm{K}}}$
CoCrMnFeNi	FCC [1]	3.6 [1]	2.312 [2]	7.007 [2]	0.0303	143 [3]	35.067	2.21	4.42	7.36	0.42	0.21	0.12
CoCrCuFeNi	FCC [4]	3.602 [4]	2.284 [2]	6.721 [2]	0.0294	156 [5]	37.792	2.10	4.20	7.00	0.41	0.21	0.12
AlCoCrFeNi	BCC [6]	2.874 [6]	2.34 [2]	9.466 [2]	0.0405	150 [7]	37.229	2.03	4.06	6.77	0.81	0.40	0.24

Table S2: Parameters for several equiatomic HEAs, where a is the lattice parameter, $\bar{l}$ is the mean nearest neighbor lengths, Σ is the standard deviation of nearest-neighbor lengths, K_2 is the coefficient of the bond stiffness estimated as $3B\bar{l}/2\sqrt{2}$, and $T_{\mathcal{T}}$ is the reduced temperature corresponding to the actual temperature $\mathcal{T}$.

7 Method

7.1 Comparison of distributions of nearest-neighbor and second nearest-neighbor lengths.

The comparison of distributions of nearest-neighbor and second nearest-neighbor lengths at 0 K with data from DFT calculations [8] is shown in Fig. S18.

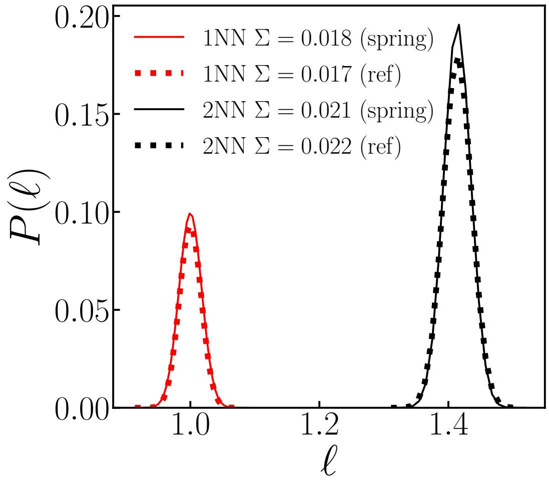

Figure S18: Comparison of distributions of nearest-neighbor and second nearest-neighbor lengths at 0 K with data from DFT calculations (Extreme Mechanics Letters, 2017, 11, 84-88).

7.2 Random, harmonic-bond HCP&BCC crystal.

The data for the MD simulations of random, harmonic-bond BCC crystal are shown in Fig. S19.

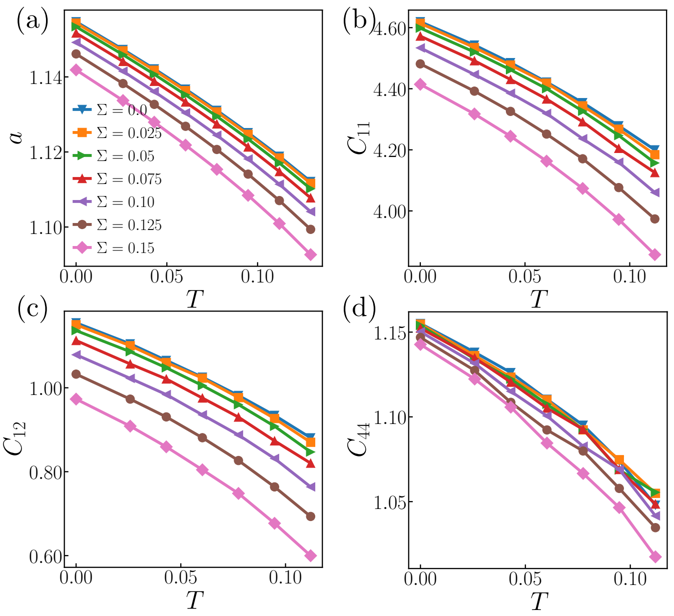

Figure S19: MD results for random, harmonic-bond BCC crystal including 2NNs. (a) The lattice parameter vs. temperature with various randomness Σ. (b), (c) and (d): The cubic elastic constants vs. temperature with various randomness Σ, sharing same legend in (a).

The data for the MD simulations of random, harmonic-bond HCP crystal are shown in Fig. S20.

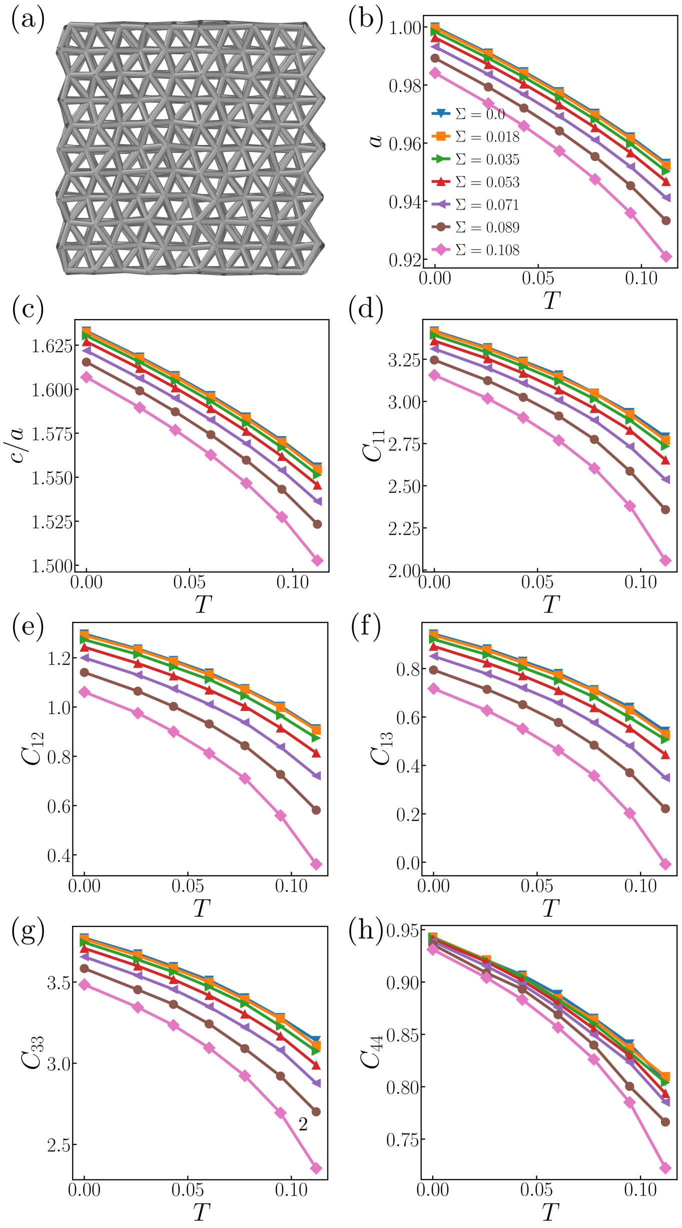

Figure S20: MD results for random, harmonic-bond hcp crystal. (a)(b) The lattice parameter a and c/a vs. temperature with various randomness Σ. (c)(d)(e)(f)(g): The cubic elastic constants vs. temperature with various randomness Σ, sharing same legend in (a).

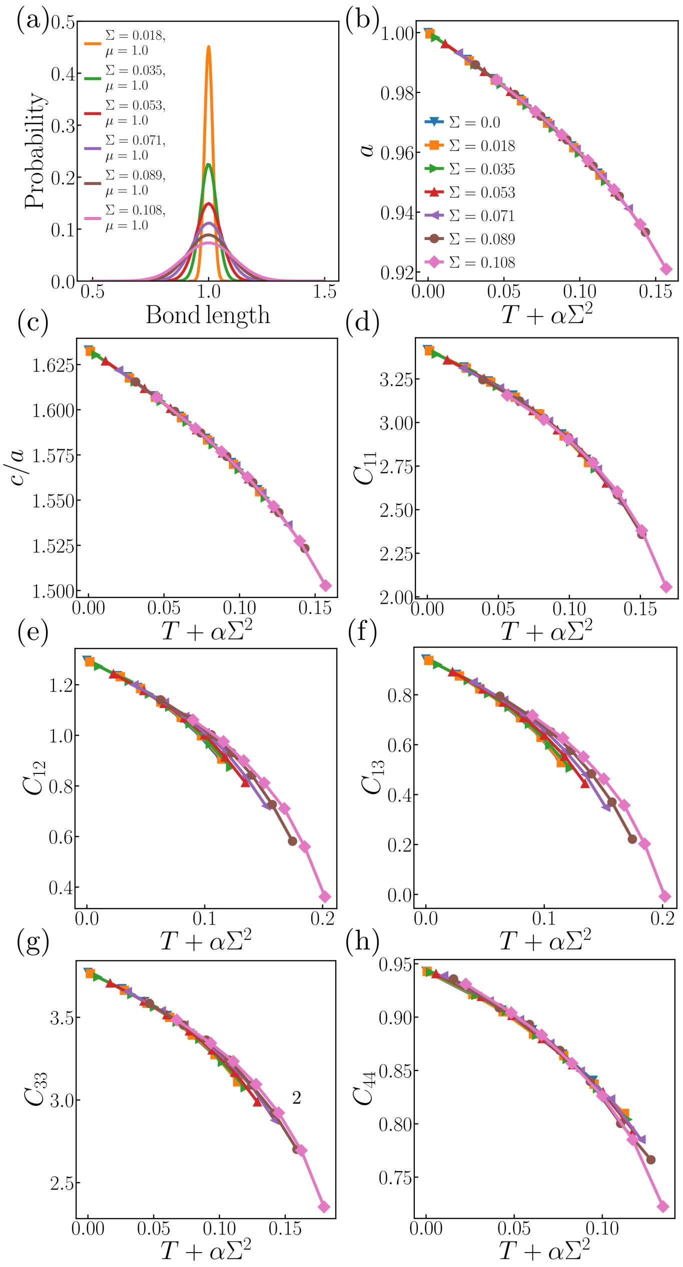

Figure S21: Replot of Fig. S20 with the horizontal axes changed to $T + \alpha\Sigma^2$. The best fit values of α are 2.0, 2.0, 2.5, 4.0, 4.0, 3.0, and 1.0 for a, c/a, C_{11}, C_{12}, C_{13}, C_{33}, and C_{44}, respectively

7.3 Random, harmonic-bond stiffness FCC crystal.

The data for the MD simulations of random, harmonic-bond stiffness FCC crystal are shown in Fig. S22.

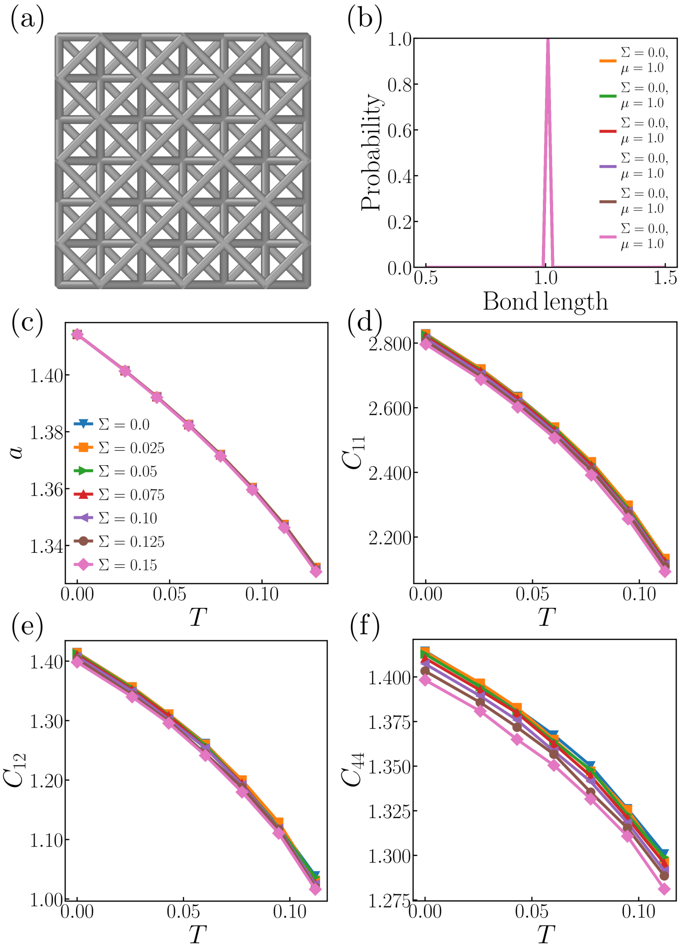

Figure S22: MD results for random, harmonic-bond stiffness FCC crystal. (a) The lattice parameter vs. temperature with various randomness Σ. (b), (c) and (d): The cubic elastic constants vs. temperature with various randomness Σ, sharing same legend in (a).

7.4 Random, EAM potential FCC crystal.

The data for the MD simulations of random, EAM potential [9] FCC crystal are shown in Figs. S23 and S24.

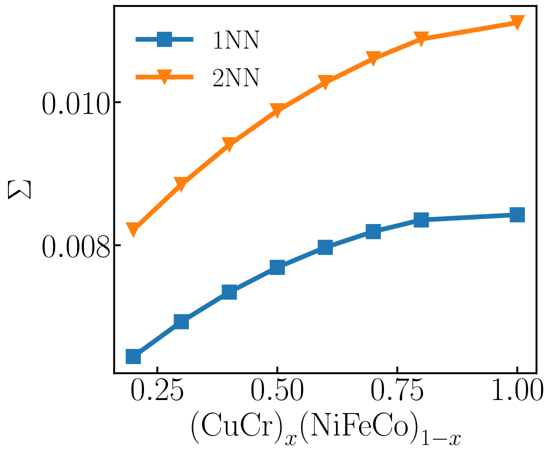

Figure S23: The standard deviation of the nearest-neighbor distance Σ vs. the composition x in the formula $(CuCr)_x(NiFeCo)_{1-x}$, obtained by the MD simulation with an EAM potential for FCC HEAs.

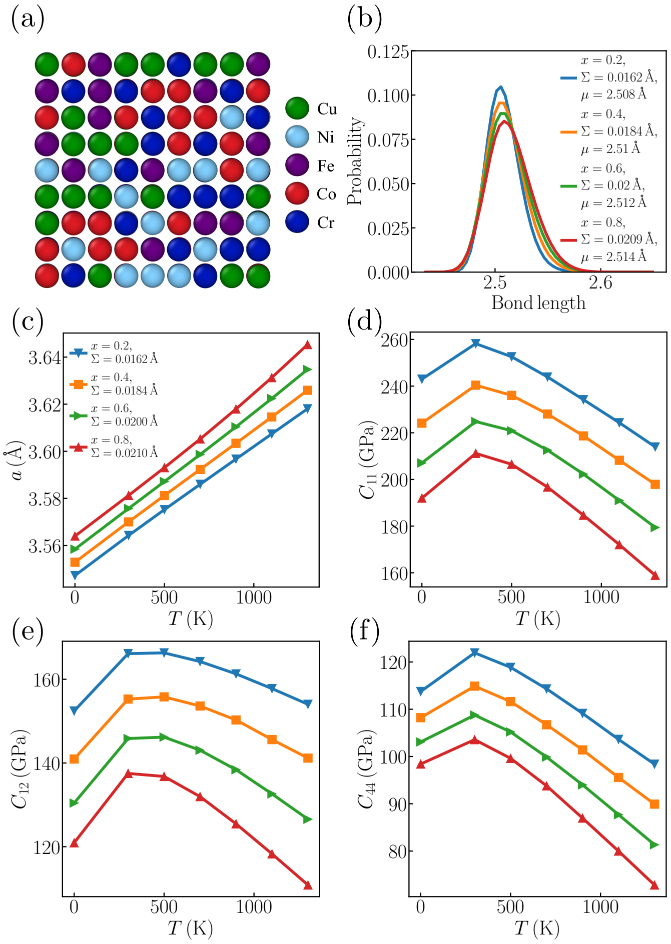

Figure S24: MD results for random, EAM potential FCC crystal. (a) lattice parameter and (b)(c)(d) C11 , C12 , C44 elastic constants versus effective temperature for various degrees of bond length randomness std., as indicated in legend, for the FCC CuNiFeCoCr crystal [9] with various contents.

7.5 Born criterion for stability of FCC/BCC/HCP

Table S3: Stability of FCC/BCC/HCP harmonic bond models with nearest- and second nearest-neighbors considered by Born criterion based on MD simulations. Elastic constants are normalized.

	neighbor	C_{11}	C_{12}	C_{44}	$C_{11} - C_{12}$	C_{44}	$C_{11} + 2C_{12}$	Stability
FCC	1NN	2.82868	1.41449	1.41439	> 0	> 0	> 0	Stable
	2NN	8.48622	1.41446	1.41483	> 0	> 0	> 0	Stable
BCC	1NN	1.15487	1.15487	1.15486	$= 0$	> 0	> 0	Unstable
	2NN	4.61935	1.15487	1.15507	> 0	> 0	> 0	Stable
	neighbor	C_{11}	C_{12}	C_{44}	$C_{11} - C_{12}$	C_{44}	$(C_{11} + 2C_{12})C_{33} - 2C_{13}^2$	Stability
HCP	1NN	3.41805	1.29659	0.94298	> 0	> 0	> 0	Stable

References

[1] F. Otto, A. Dlouhý, K. G. Pradeep, M. Kuběnová, D. Raabe, G. Eggeler, and E. P. George, Decomposition of the single-phase high-entropy alloy CrMnFeCoNi after prolonged anneals at intermediate temperatures, Acta Materialia **112**, 40 (2016).

[2] P. Pyykkö and M. Atsumi, Molecular single-bond covalent radii for elements 1–118, Chemistry–A European Journal **15**, 186 (2009).

[3] A. Haglund, M. Koehler, D. Catoor, E. P. George, and V. Keppens, Polycrystalline elastic moduli of a high-entropy alloy at cryogenic temperatures, Intermetallics **58**, 62 (2015).

[4] S. Praveen, B. Murty, and R. S. Kottada, Phase evolution and densification behavior of nanocrystalline multicomponent high entropy alloys during spark plasma sintering, JOM **65**, 1797 (2013).

[5] C. Li, R. Dedoncker, L. Li, F. Sedghgooya, F. Zighem, V. Ji, D. Depla, P. Djemia, and D. Faurie, Mechanical properties of CoCrCuFeNi multi-principal element alloy thin films on kapton substrates, Surface and Coatings Technology **402**, 126474 (2020).

[6] E. Strumza and S. Hayun, Comprehensive study of phase transitions in equiatomic AlCoCrFeNi high-entropy alloy, Journal of Alloys and Compounds **856**, 158220 (2021).

[7] B. Cheng, F. Zhang, H. Lou, X. Chen, P. K. Liaw, J. Yan, Z. Zeng, Y. Ding, and Q. Zeng, Pressure-induced phase transition in the AlCoCrFeNi high-entropy alloy, Scripta Materialia **161**, 88 (2019).

[8] S. Liu and Y. Wei, The Gaussian distribution of lattice size and atomic level heterogeneity in high entropy alloys, Extreme Mechanics Letters **11**, 84 (2017).

[9] D. Farkas and A. Caro, Model interatomic potentials and lattice strain in a high-entropy alloy, Journal of Materials Research **33**, 3218 (2018).
